# Probabilistic Evaluation of Drought in CMIP6 Simulations

**DOI:** 10.1029/2021EF002150

**Published:** 2021-10-11

**Authors:** Simon Michael Papalexiou, Chandra Rupa Rajulapati, Konstantinos M. Andreadis, Efi Foufoula‐Georgiou, Martyn P. Clark, Kevin E. Trenberth

**Affiliations:** ^1^ Department of Civil Engineering University of Calgary Calgary AB Canada; ^2^ Global Institute for Water Security University of Saskatchewan Saskatoon SK Canada; ^3^ Faculty of Environmental Sciences Czech University of Life Sciences Prague Prague Czech Republic; ^4^ Centre for Hydrology University of Saskatchewan Saskatoon SK Canada; ^5^ Department of Civil and Environmental Engineering University of Massachusetts Amherst Amherst MA USA; ^6^ Department of Civil and Environmental Engineering University of California, Irvine Irvine CA USA; ^7^ Department of Earth System Science University of California, Irvine Irvine CA USA; ^8^ National Center for Atmospheric Research Boulder CO USA

**Keywords:** CMIP6, droughts, reliability of climate models, climate change, Hellinger distance, precipitation

## Abstract

As droughts have widespread social and ecological impacts, it is critical to develop long‐term adaptation and mitigation strategies to reduce drought vulnerability. Climate models are important in quantifying drought changes. Here, we assess the ability of 285 CMIP6 historical simulations, from 17 models, to reproduce drought duration and severity in three observational data sets using the Standardized Precipitation Index (SPI). We used summary statistics beyond the mean and standard deviation, and devised a novel probabilistic framework, based on the Hellinger distance, to quantify the difference between observed and simulated drought characteristics. Results show that many simulations have less than ±10% error in reproducing the observed drought summary statistics. The hypothesis that simulations and observations are described by the same distribution cannot be rejected for more than 80% of the grids based on our H distance framework. No single model stood out as demonstrating consistently better performance over large regions of the globe. The variance in drought statistics among the simulations is higher in the tropics compared to other latitudinal zones. Though the models capture the characteristics of dry spells well, there is considerable bias in low precipitation values. Good model performance in terms of SPI does not imply good performance in simulating low precipitation. Our study emphasizes the need to probabilistically evaluate climate model simulations in order to both pinpoint model weaknesses and identify a subset of best‐performing models that are useful for impact assessments.

## Key Points

- Simulations reproduce observed drought duration and severity in terms of the Standardized Precipitation Index (SPI) but simulated low precipitation is considerably biased
- Variance in drought statistics among the simulations is higher in the tropics compared to other latitudinal zones
- Good model performance in terms of SPI does not imply that low precipitation values are well simulated by the climate models

## 1 Introduction

About half of the Earth’s land area is drought prone (Kogan, 1997) and an average of 55 million people around the world are affected by drought every year (Vatter et al., 2019). During 1900–2011, more than 11 million people lost their lives and two billion people were affected by drought (Spinoni et al., 2014). Droughts may further trigger other calamities such as wildfires and heatwaves (LeComte, 2011; Sivakumar, 2005). Unlike other disasters, such as floods or earthquakes, drought is a slowly developing phenomenon and often shows persistent consequences even after the drought has ended (Vogt et al., 2011). Furthermore, due to climate change and rising temperatures, droughts are expected to increase in terms of both frequency and duration (Markonis et al., 2021; Spinoni et al., 2014; Trenberth et al., 2014; M. Zhang et al., 2013). Given the severity of impacts on agriculture, health, and ecosystems, it is important to understand drought characteristics under a changing climate.

The Coupled Model Intercomparison Project (CMIP) models simulate the spatiotemporal evolution of ocean, land, and atmospheric processes in order to understand climate variability and change (Eyring et al., 2016, 2019). In particular, these models are used extensively to understand hydroclimatic changes at regional and global scales (e.g., Janssen et al., 2016; Knutti & Sedláček, 2013; Navarro‐Racines et al., 2020; Rajczak & Schär, 2017; Toreti & Naveau, 2015). Although there is a general agreement on the wet and dry spells between observations and climate model simulations, considerable differences are noted in the precipitation extremes (Abdelmoaty et al., 2021; Chou et al., 2013; Kharin et al., 2013; H. Zhang et al., 2013). Moreover, it is challenging for climate models to represent variability in observed precipitation at sub‐seasonal, seasonal, decadal, and multi‐decadal time scales (Ault et al., 2012; Gaetani & Mohino, 2013; Joshi & Kucharski, 2017; Mann et al., 2020). More generally, precipitation characteristics (frequency, intensity, duration, type, and amount) are not well replicated in climate models (e.g., Trenberth et al., 2017). At daily and sub‐daily time scales, it is common for model‐derived precipitation to occur far too often at low rates and not enough at intense rates.

Previous versions of CMIP have been extensively used to study droughts for both historical and future climate at regional and global scales (Dai, 2013; Jiang et al., 2015; Lee et al., 2016; McCabe & Wolock, 2015; Naumann et al., 2018; Polade et al., 2014; Rhee & Cho, 2015; Trenberth et al., 2014; Ukkola et al., 2018). At global scales, the multi‐model ensemble mean pattern of dry days was in agreement with observations, while at regional scales considerable differences were noted (Polade et al., 2014). Additionally, some studies indicated that CMIP5 models overestimate the areas under extreme drought (Nasrollahi et al., 2015), and also underestimate low precipitation events in some regions such as central and western North America (Wuebbles et al., 2013). These simulations have also been used in drought risk management and mitigation strategy planning (Schleussner et al., 2016; Zhang & Wang, 2019). However, their efficacy depends on how accurately these models represent droughts and their characteristics. Here, we use the newly released CMIP6 historical simulations to investigate the ability of models to reproduce drought duration and severity as quantified by the Standardized Precipitation Index (SPI).

The latest phase, CMIP6, simulations have been recently used to understand the variations in drought projections (Cook et al., 2020; Wang et al., 2021; Zhai et al., 2020). Other studies attempted to identify evidence of anthropogenic factors on droughts (Chiang et al., 2021; Kam et al., 2021) and associated sea‐surface temperature anomalies (Zhang & Wu, 2021). Also, when assessing the ability of climate model simulations to reproduce observed drought characteristics, many studies only consider a single simulation for each climate model (Abatzoglou & Rupp, 2017; Deser et al., 2014; Nasrollahi et al., 2015; Ukkola et al., 2018; Wehner et al., 2011). However, this can obscure the actual uncertainty as simulations from a given model can vary markedly even for historical simulations. This variation within ensemble members from a single climate model is mainly due to internal variability (Deser et al., 2014; Fischer et al., 2014). In this work, all available simulations are considered from each CMIP6 model to account for both inter‐ and intra‐model variability in reproducing drought characteristics.

Several drought indices are defined in the literature (Mishra & Singh, 2010); here we use the SPI which is used to track meteorological drought. The key idea of the SPI is transforming raw precipitation data into standard normal variates and examining the dry spells (droughts) where time‐averaged SPI values are below a specific threshold. However, SPI has several limitations. In particular, the SPI does not consider evapotranspiration; this limits its applicability in quantifying future drought changes since evapotranspiration is expected to increase in the future. Finally, when comparing model simulations with observations the SPI does not account for the actual differences in the statistical properties of precipitation, that is, two time series might notably differ but could result in very similar SPI values. Despite these limitations, the SPI is the most popular drought index as it is simple to calculate using only precipitation data, and effective in analyzing wet/dry periods (Adarsh & Reddy, 2019; Hayes et al., 2011; Kumar et al., 2009; WMO & GWP, 2016). Moreover, the SPI is more comparable across different regions than other popular drought indices such as the Palmer Drought Severity Index.

The specific objectives of this study are: (a) to understand the variability of observed drought duration and severity globally, (b) to compare CMIP6 simulations’ drought duration and severity with observations, (c) to employ an easy‐to‐use measure for quantifying differences between observed and simulated drought duration distributions, and (d) identify the models that best reproduce drought characteristic in each geographical region.

## 2 Data and Methods

### 2.1 CMIP6 and Observations

Historical simulations of monthly precipitation are obtained from the World Climate Research Program (WCRP) CMIP6 data archive. The historical experiment, covering the period 1850–2014, provides simulations based on observed natural and anthropogenic forcings (Eyring et al., 2016). To include the uncertainty due to both different model physics and internal variability, we select simulations from 17 modeling groups each with more than five ensemble members, summing to a total of 285 runs (see Tables 1 and S1). Note that we use the terms “simulation” and “run” interchangeably referring to an individual ensemble member from a specific model. The spatial resolution of the models varies from 0.5 to 2.5°, therefore for consistency all simulations were regridded to a common resolution of 2°×2° using the first‐order conservative method (Jones, 1999). This resolution was selected as it is close to the average resolution of all products (approximately 1.78°), and is also in accordance with many other CMIP studies (Chou et al., 2013; Hao et al., 2013; Nguyen et al., 2017; H. Zhang et al., 2013). Clearly, the statistical properties of precipitation vary across different spatiotemporal scales (see e.g., Trenberth & Zhang, 2018) and regridding might affect the results. Yet without a common spatiotemporal resolution it is not feasible to compare models and observations.

**Table 1:** CMIP6 Models Used in This Study

CMIP6 model	Institute	No. of runs	No. of grids (lat × lon)	Reference
CESM2	National Center for Atmospheric Research, Boulder, USA	11	192 × 288	Danabasoglu (2019)
CNRM‐CM6‐1	Center National de Recherches M´et´eorologiques (CNRM); Center Europ´een de Recherches et de Formation vanc´eeen Calcul Scientifique	28	128 × 256	Voldoire et al. (2019)
CNRM‐ESM2‐1		9	128 × 256	Seferian (2018)
CanESM5	Canadian Center for Climate Modeling and Analysis, Environment and Climate Change Canada, BC, Canada	50	64 × 128	Swart et al. (2019)
E3SM‐1‐0	Lawrence Livermore National Laboratory, Livermore, USA	5	180 × 360	Bader et al. (2019)
EC‐Earth3	Consortium of various institutions from Spain, Italy, Denmark, Finland, Germany, Ireland, Portugal, Netherlands, Norway, UK, Belgium, and Sweden	8	256 × 512	EC Earth (2019)
GISS‐E2‐1‐G	NASA Goddard Institute for Space Studies, New York, USA	23	90 × 144	NASA/GISS (2018a)
GISS‐E2‐1‐H		21	90 × 144	NASA/GISS (2018b)
INM‐CM5‐0	Institute for Numerical Mathematics, Russian Academy of Science, Moscow, Russia	10	120 × 180	Volodin et al. (2019)
IPSL‐CM6A‐LR	Institut Pierre Simon Laplace, Paris, France	32	143 × 144	Boucher et al. (2018)
MIROC6	Japan Agency for Marine‐Earth Science and Technology, Atmosphere and Ocean Research Institute, National Institute for Environmental Studies, and RIKEN Center for Computational Science, Japan	10	128 × 256	Tatebe and Watanabe (2018)
MPI‐ESM1‐2‐HR	Max Planck Institute for Meteorology, Germany	10	192 × 384	Jungclaus et al. (2019)
MPI‐ESM1‐2‐LR		10	96 × 192	Wieners et al. (2019)
MRI‐ESM2‐0	Meteorological Research Institute, Tsukuba, Japan	6	160 × 320	Yukimoto et al. (2019)
NESM3	Nanjing University of Information Science and Technology, Nanjing, China	5	96 × 192	Cao and Wang (2019)
NorCPM1	NorESM Climate modeling Consortium, Norway	30	96 × 144	Bethke et al. (2019)
UKESM1‐0‐LL	Met Office Hadley Center	17	144 × 192	Y. Tang et al. (2019)

*Note*. Models with more than five simulations available at the time of the analysis are selected.

Three observational data sets are used to consider uncertainty in observations: (a) the Climate Research Unit (CRU) TS4.03 data set, calculated from daily or sub‐daily data, is derived from meteorological stations (Harris & Jones, 2017; Harris et al., 2014). The station data are gridded using the angular‐distance weighting (ADW) interpolation. Monthly precipitation over land areas, excluding Antarctica, for the period 1901–2018, at 0.5°×0.5° degree is available. (b) The Global Precipitation Climatology Center (GPCC) version 2018 data set is spatially interpolated from station data (from ∼6,000 before 1,900 to more than 50,000 stations in 2018) by using a modified version of the robust empirical interpolation method (Becker et al., 2013; Schneider et al., 2011). The GPCC has the highest temporal coverage from 1891 to 2016 and is one of the largest land‐based monthly precipitation data sets. (c) The University of Delaware (UDel) v5.01 data set, interpolated from various observational network stations including GHCN (Global Historical Climatology Network), uses climatologically aided interpolation to estimate monthly total precipitation fields (Willmott & Matsuura, 2001; Willmott & Robeson, 1995); UDel is available from 1900 to 2017. We stress that the actual observational uncertainty cannot be fully quantified even if all available data sets were used since most of them rely on the same raw data (e.g., stations) or combine data from different sources and products (see e.g., G. Tang et al., 2020, 2021). Trenberth et al. (2014) evaluated several observational data sets and noted the dependence on the number of stations, so that results could be quite different. Although the data are available for over a century, we noted irregularities (e.g., artificial cyclicity, large number of missing values, etc.) in precipitation in some grids for the earlier part of the record, mostly in Africa, South America, and some parts of Asia. Therefore, we considered the recent half century (1963–2014) when reliable data are available and warming trends have been shown to impact precipitation and temperature extremes (see e.g., Papalexiou & Montanari, 2019; Papalexiou et al., 2018).

### 2.2 Methods

**Figure 1:**
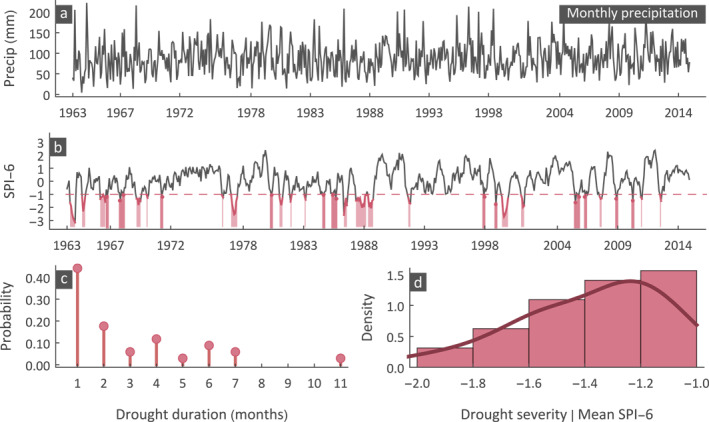
Calculating drought duration and severity using SPI‐6 for a sample precipitation time series. Plots show (a) monthly precipitation time series, (b) SPI‐6 time series highlighting the drought periods, (c) the probability distribution of drought duration, and (d) the probability distribution of drought severity.

The SPI is one of the most widely used indices to characterize meteorological drought (McKee et al., 1993) by identifying periods of precipitation deficit or excess. The precipitation monthly time series (Figure 1a) are typically aggregated with a pre‐defined moving window (e.g., 6 months) and transformed to a standard normal variate. This is the basic concept in calculating the SPI, and parametric and non‐parametric variations exist (Farahmand & AghaKouchak, 2015; Mishra & Singh, 2010; Vicente‐Serrano et al., 2012). The parametric method fits a suitable probability distribution, while the non‐parametric method calculates the empirical probabilities using a plotting position formula (typically the Weibull or Gringorten). The probabilities are then transformed to Z scores (standard normal variate), with negative values (i.e., SPI) indicating dry periods (Figure 1b). Here we use the parametric approach by fitting a Gamma (G) distribution to the monthly precipitation at each grid. Although alternative distributions could have been used the Gamma distribution is the most widely used for SPI calculations (Zhang & Li, 2020). The Gamma cumulative distribution function is given by

(1) $$F_{G}(x)=\frac{1}{\Gamma (\gamma )}\int_{0}^{x}\beta^{-\gamma }t^{\gamma -1}\text{exp}(-t/\beta )dt$$

where Γ(·) is the gamma function, and β and γ are the scale and shape parameters, respectively. In the case of precipitation time series with zeros (which would map to −∞ SPI), the unconditional probabilities are calculated as$\;G\left(x\right)=p_{0}+\left(1-p_{0}\right)F_{G}\left(x\right)$, where $p_{0}$ is the probability of zero precipitation. Thus, the SPI is calculated by $\text{SPI}\left(x\right)=\Phi^{-1}$(G(x)), where $\Phi^{-1}$ is the quantile function of the standard normal distribution.

We selected the SPI‐6 (6‐month SPI) to assess global drought severity and duration, as the medium‐term accumulation period of 6 months is more suitable to describe meteorological and agricultural (soil moisture) drought conditions. On the other hand, SPI‐12 and SPI‐24 are more suitable to reveal how streamflow, reservoirs, and groundwater respond to longer‐term precipitation anomalies (WMO, 2012). Note that SPI does not consider evapotranspiration but quantifies periods of low or high precipitation, thus, winter precipitation deficit may not lead in adverse drought effects due to low evapotranspiration. Following McKee et al. (1993), drought starts when the SPI falls below a threshold and ends when the threshold is exceeded. A common threshold for defining moderate drought is the 6‐month SPI≤−1 (McKee et al., 1993; WMO, 2012). The duration of a drought event is then defined as the number of months between the start and end of drought, and severity is calculated as the integral between the SPI line and the threshold line for each event (Figure 1b). From the identified drought events we can form probability distributions describing drought duration (Figure 1c) and severity (Figure 1d). The probability distribution of drought duration is discrete, while the one for severity is continuous with an upper bound at −1 when droughts are identified by SPI≤−1.

SPI was calculated using a mixed‐type distribution approach which correctly accounted for zero precipitation values. However, by definition $\text{SPI}=\Phi^{-1}\left(p_{0}+\left(1-p_{0}\right)F_{G}\left(x\right)\right)$, thus, if a time series has zeros, the SPI has a minimum at ${\text{SPI}}_{\text{min}}=\Phi^{-1}\left(p_{0}\right)$. For example, if a time series has $p_{0}>2.8\%$ or $p_{0}>15.8\%$ then SPI values will always be larger than −2 and −1, respectively, making it impossible to identify drought at those thresholds. We selected −2 as the threshold as it typically designates severe droughts. Therefore, we did not study grids having $p_{0}>2.8\%$, which occurred mostly in the desert regions of Africa.

Our objective was to assess the agreement of CMIP6 simulated drought duration and severity, in terms of SPI, against benchmark observational data sets. Note that SPI agreement between models and observations does not imply good performance of models in simulating low precipitation values, since there are strong model biases in low precipitation (see Section 3.4). Here, we compared observed and simulated summary statistics, including the mean, coefficient of variation, skewness, and maximum of duration and severity as they summarize the properties of their distributions. Given three observational products, there is a range of values for each statistic. One option is to consider the observational range in the three products as the maximum minus the minimum values of the statistic. Then one could assume that a simulation would agree if its computed summary statistic falls within the observational range. Yet a concern with this approach is that any large disagreement among the observational products could lead to artificially high agreement of simulations (since the range will be large). For example, in the desert parts of Africa, northern parts of Russia and Canada, we note a high observational range which results in all runs agreeing with observations. This stems from the disagreement among the observations and not the agreement of models with observations. Therefore, we chose a relative error threshold of 10% to designate agreement between summary statistics of the CMIP6 simulations and each observational product separately. Although any error threshold selection is subjective, a value of 10% is arguably a compromise of strictness and meaningful results. For example, if the mean drought duration predicted by a CMIP6 simulation has a relative error of less than 10%, when compared with the observational data set mean duration then that simulation run is considered in agreement. For each data set we have calculated the percentage of runs that lie within the 10% range, and finally obtained the average percentage from the three data sets. For duration, we considered all four statistics whereas for severity we examined only the coefficient of variation and skewness. Mean severity is theoretically constant for any SPI time series for a given threshold; any fluctuations noted in observations or simulations are due to sample variation.

An additional approach to evaluate the differences between probability distributions is through information measures such as the Kullback‐Leibler (KL) divergence (e.g., Nasrollahi et al., 2015; Rajsekhar et al., 2015) or the Hellinger (H) distance. Here we used the H distance (Hellinger, 1909), which conveniently ranges from 0 to 1 making it more intuitive than the KL divergence. If d is the drought duration (in months) and p(d) and q(d) are two discrete probability distributions describing the drought duration in two samples, then the H distance is given by

(2) $$H^{2}\left(p,q\right)=\frac{1}{2}\overset{d_{\text{max}}}{\underset{d=1}{\sum }}{\left(\sqrt{p(d)}-\sqrt{q(d)}\right)}^{2}$$

where $d_{\text{max}}$ is the maximum duration observed in any of the two samples. For two continuous distributions p(x) and q(x) the H distance is given by

(3) $$H^{2}\left(p,\;q\right)=\frac{1}{2}\int_{x\in X}{\left(\sqrt{p\left(x\right)}-\sqrt{q\left(x\right)}\right)}^{2}$$

where X is the continuous sample space. The H distance satisfies the property 0≤H≤1. If H distance is 0 then the distributions in question are practically identical; the larger the H distance (tending to 1) the larger the difference between the distributions. Here, we compare the duration and severity probability distributions between observations and CMIP6 simulations. For duration and severity, we used the discrete (Equation 2) and continuous (Equation 3) forms, respectively.

Nevertheless, specific values of H are not easy to interpret. For example, if the H distance is 0.3 then the question that naturally arises is whether this value indicates sufficient similarity between the two distributions or not. In other words, a specific H threshold needs to be identified below which we can assume that distributions match. In order to make the Hellinger distance interpretable and estimate this threshold, we devise a new framework based on Monte Carlo simulations. Particularly,

1. For a given observational product, the mean and standard deviation of duration values is estimated at each grid.
2. A flexible discrete distribution, that is, the Pólya‐Aeppli distribution (also known as geometric Poisson) is fitted to the data reproducing the observed mean and standard deviation.
3. Two random samples are generated from the fitted distribution having the same sample size as the observed one and the H distance is estimated. Note that although these samples are generated from the same distribution, due to sample variations the H value is always larger than zero. This depends on the sample size (the larger the sample the closer H is to zero) and the distribution parameters.
4. The previous step is repeated 1,000 times to study how the H distance varies given that the samples are generated from the same distribution. Then these 1,000 H values are used to define a 90‐th percentile threshold $H_{0.9}$. This implies that 90% of the generated samples have $H<H_{0.9}$.
5. The interpretation is that if the H distance in this specific grid between the probability distributions computed from observations and the simulation is smaller than $H_{0.9}$ then the hypothesis that the simulated and observed samples come from the same distribution cannot be rejected.

Note that this is a laborious process, as the H threshold must be identified for each grid and each observational product. Once the H threshold is defined, the percentage of climate model simulations (285 in total) with $H<H_{0.9}$ correspond to the agreement of the simulations with each observational product. The average agreement can then be estimated from the three agreement values. For drought severity, the same process can be applied by using continuous distributions; however, we skipped the previous detailed approach since the severity distributions are expected to not differ strongly since the mean severity is theoretically fixed (as the mean of a truncated standard normal distribution and approximately equal to −1.53). Moreover, it is technically far more laborious to compute H given the required numerical integration.

## 3 Results and Discussion

### 3.1 Observed Drought Duration

**Figure 2:**
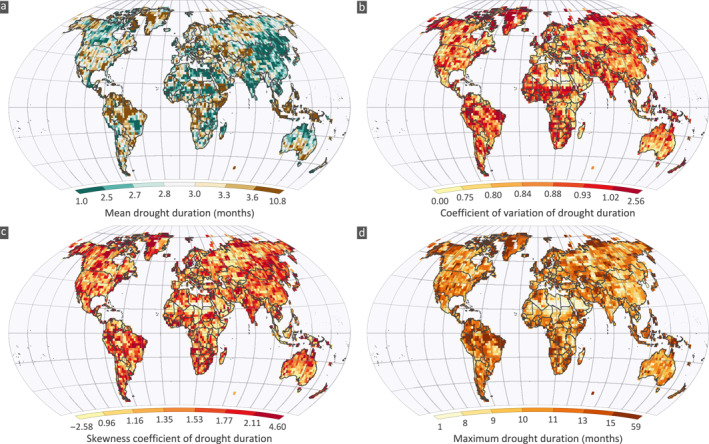
Spatial variation of observed (a) mean, (b) coefficient of variation, (c) skewness, and (d) maximum drought duration for the moderate (SPI≤−1) case during 1963–2014 for CRU (see Figures S1 and S2 for GPCC and UDel, respectively).

A relatively clear pattern of mean drought duration is evident for moderate droughts (Figure 2a for CRU, Figures S1 and S2 for GPCC and UDel, respectively) for the three observation data sets. Yet differences are observed, for example, in parts north of Canada and Russia. This could be due to deficiencies in data sources and errors in measuring precipitation at high altitudes such as wind undercatch of solid precipitation and recording of trace events (Rajulapati et al., 2020). High mean durations (>4 months) are observed in areas near the equator such as parts of Amazonia, Southwestern and eastern Africa, Indonesia and Philippines. Low mean duration (<2.7 months) is observed in India, eastern parts of Asia, northern parts of Africa and Australia, and southern parts of South America. The spatial patterns of coefficient of variation $\left(C_{V}\right)$ and skewness $\left(C_{S}\right)$ are similar to each other in all data sets (Figures 2b, 2c, S1, and S2), with high values ($C_{V}>0.9$ and $C_{S}>1.8$) observed in parts of India, mainly in Western Africa, eastern parts of Asia, northern parts of Australia and Brazil. In general, many regions exhibit an inverse relationship between the mean and coefficient of variation (i.e., grids with high mean values have low skewness and $C_{V}$). The maximum duration varies from 1 to 61 months (Figure 2d) with spots of high durations observed for example in Amazonia. Note, that we have included Greenland where values exist, these results are not reliable, and they should be discounted.

### 3.2 CMIP Agreement With Observed Drought Duration

**Figure 3:**
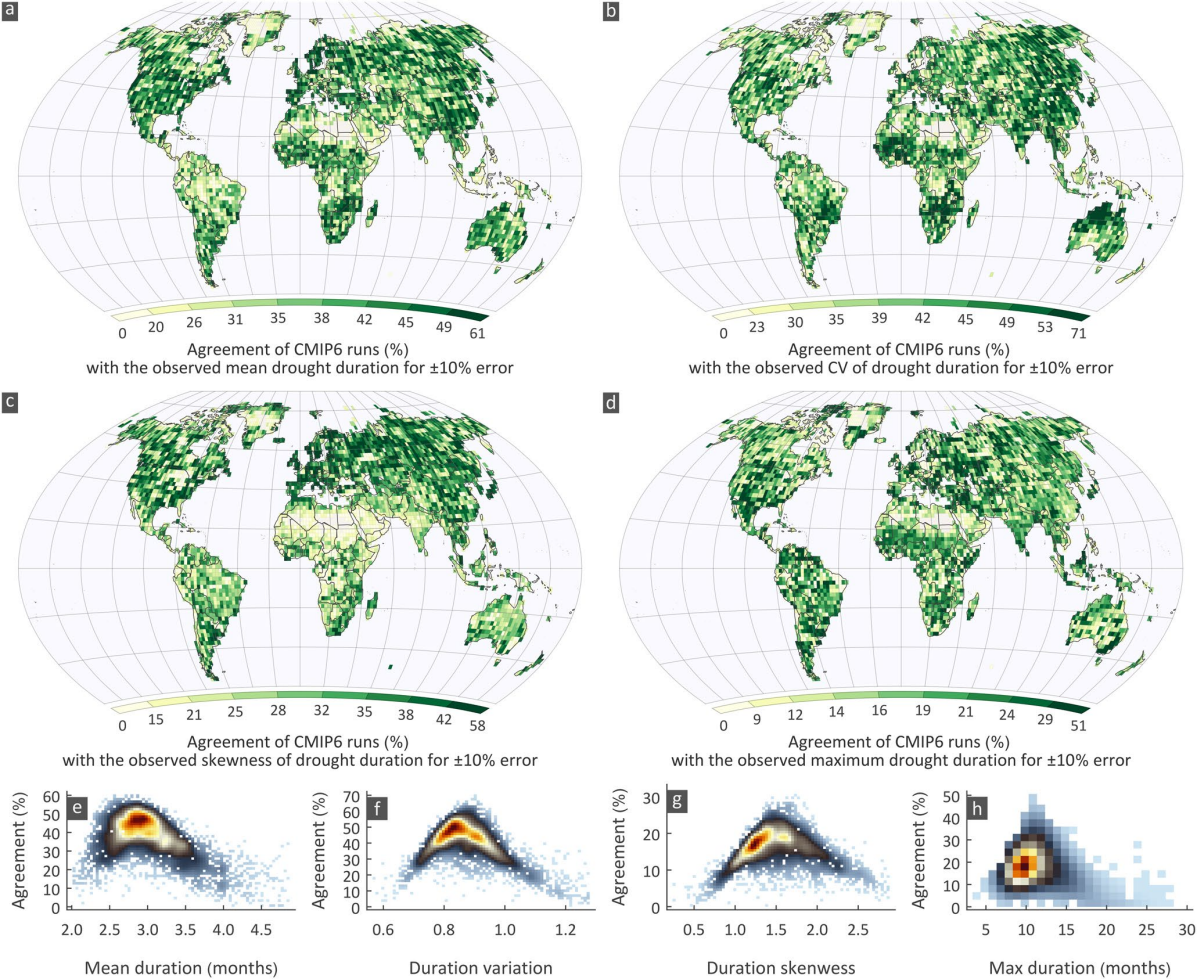
Average agreement for drought duration statistics between CMIP6 runs and the three observational data sets (CRU, GPCC and UDel) for 1963–2014. (a–d) Spatial variation of average agreement in four statistics, (e–h) agreement versus observed statistics’ values.

We compared the 285 CMIP6 runs with the observed drought duration statistics of the three observational data sets and calculated the percentage of runs within ±10% error for each statistic (see Figures S3–S5 for the agreement of drought duration statistics between CMIP6 runs and CRU, GPCC, and UDel, respectively). In general, there is no clear spatial pattern of agreement in the four statistics examined, that is, large regions with consistently high (or low) agreement are not observed. Yet these “noisy” patterns match fairly well among the three data sets except at a few locations. The average percentage of runs within ±10% error for each statistic shows a strong spatial variation (Figure 3). In general, there is a large percentage of runs for most grids that agree with the observed mean drought duration; 50% of grids have more than 36% agreement (Figure 3a). For the variation (Figure 3b) the agreement is also high with 50% of grids having more than 40% agreement. For the skewness and maximum duration, the agreement is lower (50% of grids have more than 16% and 33% average agreement; Figures 3c and 3d), yet this is anticipated as the sample variability of the skewness and maximum is large.

The analysis reveals that the average value of a statistic is reproduced well by the ensemble, including the skewness and maximum duration. However, grids with low or high values of the statistic have low agreement; this indicates that only a few CMIP6 runs reproduce extreme values of the observed summary statistics of drought duration. For example, the agreement in grids with low (<2.47 months corresponding to the 10‐th percentile) and high (>3.77 months; 90‐th percentile) mean duration is on average 16% and 5% respectively; in contrast, grids with values ranging from 2.6 to 3.3 months, corresponding to the central 50% of values (interquartile range), have on average 50% agreement. Similarly, in the other statistics the highest agreement is observed around their mean, for example the agreement for values ranging within the interquartile range is 42%, 40%, and 39% for the variation, skewness, and maximum, respectively. This is apparent in the scatterplots showing the agreement *vs*. the statistics' values (Figures 3e–3h). The peak agreement occurs within a narrow range (dark red region in Figures 3e–3h) showing that most runs are good in simulating the common statistical values in the observations.

**Figure 4:**
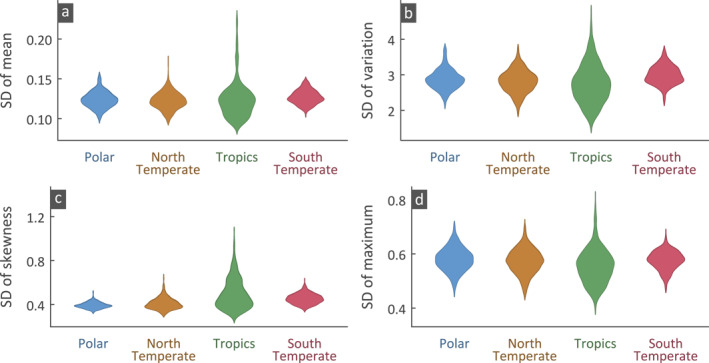
The standard deviation (SD) of a statistic among the 285 simulations is estimated in each grid; then all estimated SDs in each latitudinal zone are depicted as violin plots: (a) mean, (b) coefficient of variation, (c) skewness, and (d) maximum of drought durations.

We also examined the variability of drought characteristics among climate models at different latitudes. At each grid we estimate the standard deviation of each statistic among the 285 models for four latitudinal zones, that is, north polar (90°−66.5°N), north temperate (66.5°−23.5°N), tropical (23.5°N−23.5°S), and south temperate (23.5°−66.5°S). Violin plots (Figure 4) formed from the standard deviations of the grids in each zone (584, 1,956, 1,074, and 259 grids for north polar, north temperate, tropics and south temperate zones, respectively) show that the tropical grids have a much larger spread compared to the other regions. Specifically, the north polar region has low variability among the climate models. However, for observations, this variability is almost the same in all zones (Figure S6).

**Figure 5:**
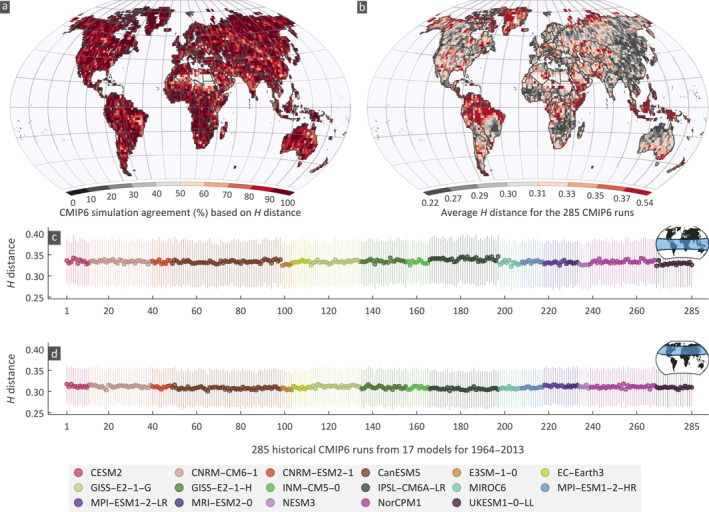
Percentage agreement between simulated and observed drought statistics based on Hellinger (H) distance and average H‐distances over the globe and different latitudinal zones. (a) The average percentage agreement of CMIP6 simulations with the three observations (CRU, GPCC, and UDel) based on H distance for the moderate drought duration, (b) spatial variability of average distance globally, (c and d) H distance for the tropical (23.5°N−23.5°S) and north temperate (66.5°−23.5°N) zones respectively; Lines represent 50% central Hdistance values. For individual data set (CRU, GPCC, and UDel) results see Figures S7–S9.

Apart from investigating individual statistics we used the Hellinger distance as an overall measure to quantify the similarity between the simulated and observed drought duration probability distributions. Here we estimated the H distance using the three observation data sets for each simulation and at each grid (Figure S7). Then, the percentage of simulations that agree with an observation data set is calculated by considering the H values of simulations that are below the threshold ($H_{0.9}$) obtained from the Monte Carlo simulations. Finally, the average percentage of agreement at each grid is calculated using the agreement obtained from the three observational data sets (Figure 5a). There is at least one simulation that agrees ($H<H_{0.9}$) with the observations in all the grids. One could assume that the models are simulating the drought duration distribution correctly as we cannot reject the hypothesis that the distributions are similar based on the H distance. Such a conclusion might be misleading due to inference limitations stemming from the small sample sizes. In essence, it is statistically hard to assess if the distributions between two small‐size samples match or not. The ±10% error analysis (Figure 3) shows a much smaller agreement compared to the *H*‐distance agreement (Figure 5) as the ±10% error is a stricter measure.

The average H distance of the 285 simulations with the three observation sets (3×285H values averaged) (Figure 5b; for individual observation data sets see Figures S8–S10) has similar spatial patterns among the three observations. Clusters of high H distance (>0.4) are spotted in the Alaska, northern Russia and Canada, southern South America and southeastern Australia. High H values are also observed in tropical regions, indicating that models have a considerable difference with observations. Globally, among the runs, there is no huge variability of the H distance; yet we noted a considerable variation in the tropical zone (23.5°N−23.5°S; whiskers in Figure 5c) compared to the north temperate zone (66.5°−23.5°N; Figure 5d).

**Figure 6:**
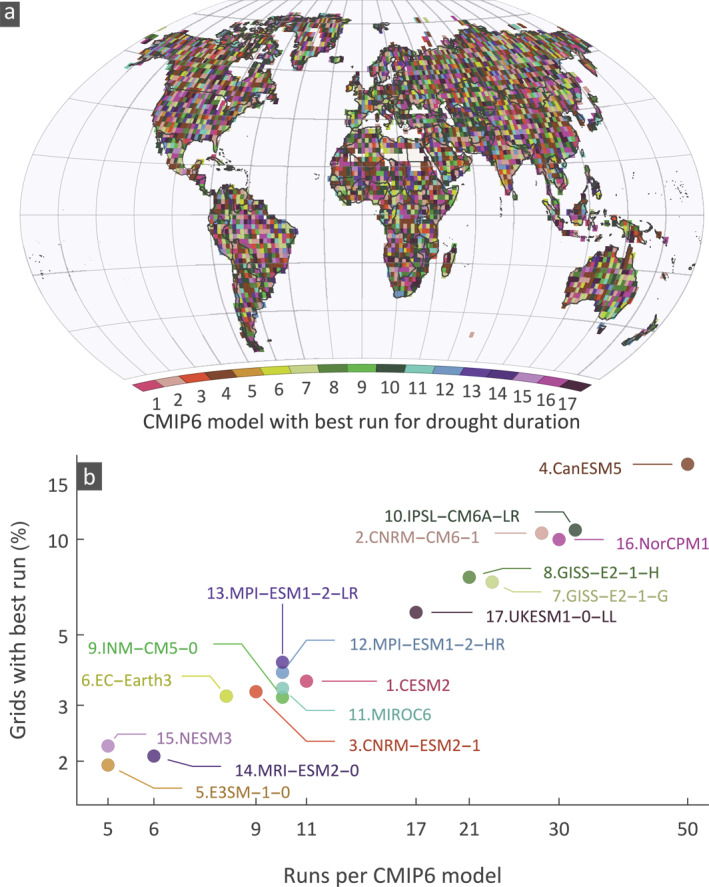
(a) Spatial variability of CMIP6 models corresponding to the best performing run according to the minimum average Hellinger (H) distance; the H distance for all three data sets is averaged; (b) relationship between the number of CMIP6 runs per model and the average percentage of grids where runs of the model perform best.

The spatial distribution of the best performing models does not show any discernible pattern (Figure 6a). For each grid, the best performing model was identified as the one with the minimum average H distance for one of its runs. It appears that all models have grids where their runs perform best, with the number of grids that a model was considered best following an almost linear relationship with the number of runs in each model (Figure 6b). For example, the CanESM5 has the highest number of runs (50) and the highest percentage (17%) of grids that perform best. There is no single simulation among the 50 CanESM5 simulations that is markedly better; the highest percentage is 3.33% for the simulation r19i1p1f1 (see Figure S11 for the percentage of grids with best CanESM5 model spread among the 50 runs). This suggests that is difficult to assess which model is better, as the dependence on the number of runs obfuscates the skill of the model itself.

### 3.3 Drought Severity

**Figure 7:**
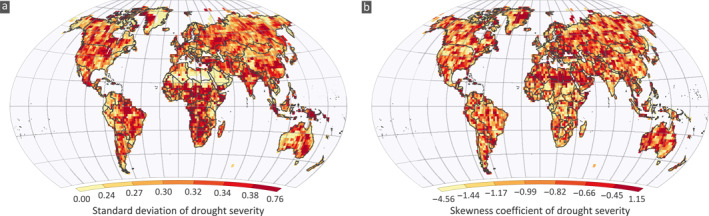
Spatial variability of (a) observed standard deviation, (b) observed skewness of drought severity for the CRU data set (see Figures S11 and S12 for GPCC and UDEL, respectively).

**Figure 8:**
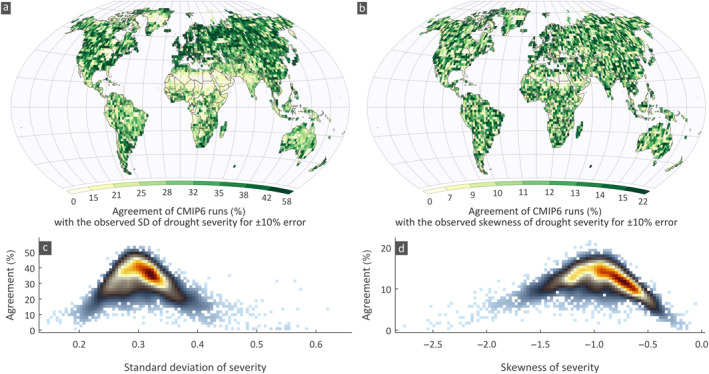
Average percentage agreement of CMIP6 simulations with the observed (a) Standard Deviation (SD) and (b) skewness of drought severity. The percentage agreement for each data set SD and skewness is averaged. Scatter plots between the average percentage agreement of CMIP6 simulations and average observed (c) SD (d) skewness of severity over all land areas for moderate droughts.

Drought severity expresses the deficit below the threshold for the duration of a drought event and is important for interpreting how intense the drought is during the event. We computed the standard deviation (SD) and skewness of severity, as the mean severity is theoretically constant according to the definition of the SPI. Quantifying differences in these two statistics between observations and simulations is also crucial as they reveal the probabilistic profile of severity indicating how large variations should be expected from the mean behavior. This, for example, shows the tendency of extreme severity in the CMIP6 runs compared to the observations. The variability of SD and skewness for the CRU shows coherent spatial patterns (Figure 7; see Figures S12 and S13 for GPCC and UDel, respectively). We noted a relatively high SD in CRU compared to UDel and GPCC. Spatially, the patterns are similar for the observational data sets. The SD of severity in observations (Figure 8a) is lower compared to CMIP6 simulations. Spatially, high SD (>0.38 mm/month; 90‐th percentile) is observed in equatorial and southern Africa, northern India, eastern grids of South America, northern parts of US and Canada and eastern Australia. Low SD (<0.25 mm/month; 10‐th percentile) is found mostly in the desert parts of Africa, middle east countries, rangelands of Australia, most parts of Alaska and north western coast of South America. Interestingly, the observed patterns of SD and skewness (Figure 8b) are in general contrasting, with regions having high SD indicating low skewness and vice versa (cross correlation is −0.44,−0.39, and −0.42 for CRU, GPCC, and UDel, respectively). The CMIP6 simulations also reproduce this behavior very well as the cross‐correlation ranges from −0.33 to −0.59, with an average value equal to −0.45.

The percentage of simulations within ±10% error of the observed SD and skewness for the three observational data sets and the average percentage (Figure 8; for CRU, GPCC, and UDel see Figures S14–S16) feature high agreement in SD compared to skewness (yet as aforementioned this is anticipated as the skewness has higher variability); about 30% and 11% agreement is noted in the SD and skewness respectively for 50% of grids. We did not observe any spatial coherence in the agreement between the statistics examined. Similar to the duration, runs simulate well values varying close to the observed mean of the statistic, in contrast to low and high values (Figures 8c and 8d). The agreement in grids having low, high and values ranging within the interquartile range of the SD is on average 17%, 9% and 44%, respectively. For low ($C_{S}<-1.45$; 10‐th percentile), high ($C_{S}<-0.56$; 90‐th percentile), and the central 50% ($-1.19<C_{S}<-0.73$) of the skewness the average agreement is 13%, 19%, and 39%, respectively.

Note that among the 3,873 grids, for SD, zero agreement is observed only for 84, 77, and 53 grids for the CRU, GPCC, and UDel, respectively (for skewness zero agreement is observed in 87, 104 and 97 grids). This implies that for the most grids and regions there is at least one CMIP6 run that reproduces very well the severity properties.

The mean H distance for severity is 0.161, 0.157 and 0.16 (Figure S16), respectively, for the CRU, GPCC, and UDel data sets. More than 85% of simulations have an H value less than 0.25 globally. This is expected as, by definition, the mean value of severity is theoretically the same. On the other hand, if one considers total severity, considerable differences among the simulations and observations could be noted with high H distances. However, since the total severity is related to the duration, the differences noted in the duration are expected to be observed in the total severity as well.

### 3.4 Biases in Low Precipitation

As previously mentioned, good model performance in terms of SPI does not imply good model performance in simulating low precipitation values. For example, two time series can have the same SPI values while completely differ in their magnitudes (e.g., a time series and any rescaled version of it). A detailed bias evaluation of precipitation is outside of the study’s scope; however, to make the previous point clear and avoid any misinterpretation of the SPI results we show the bias in low monthly precipitation.

We assess the model bias in monthly precipitation that corresponds to the 15‐th percentile; this percentile is representative of low precipitation (see e.g., Ukkola et al., 2020). Note that fixing an absolute value as a drought threshold is not practical for a global analysis. For example, the 15‐th percentile of CRU values in all grids is approximately 4 mm/month. If such a fixed threshold is used globally, then 23% of the grids do not have values below this threshold, 50% of girds have less than 20 values, and in contrast, this threshold corresponds to a value above the median for 10% of grids (and thus this cannot be considered low precipitation). In each grid the 15‐th percentile precipitation is estimated in observations (Figure S18) and simulations and the bias is calculated as the difference between the observed and simulated value (Figure S19). Note, that no major difference is noted in the low precipitation among the three data sets (except in Greenland where data are not reliable enough to draw conclusions; Figure S18). Given the similarity in low precipitation among the three data sets, we have considered only the CRU data set for further analysis. Considerable biases are noted in the ensemble mean of each model with the CRU values (Figure S19). Particularly in some grids (e.g., in eastern Asia) models simulate values higher by more than 600 mm/month while in other regions (e.g., in Amazonia) models simulate values up to 300 mm/month less.

**Figure 9:**
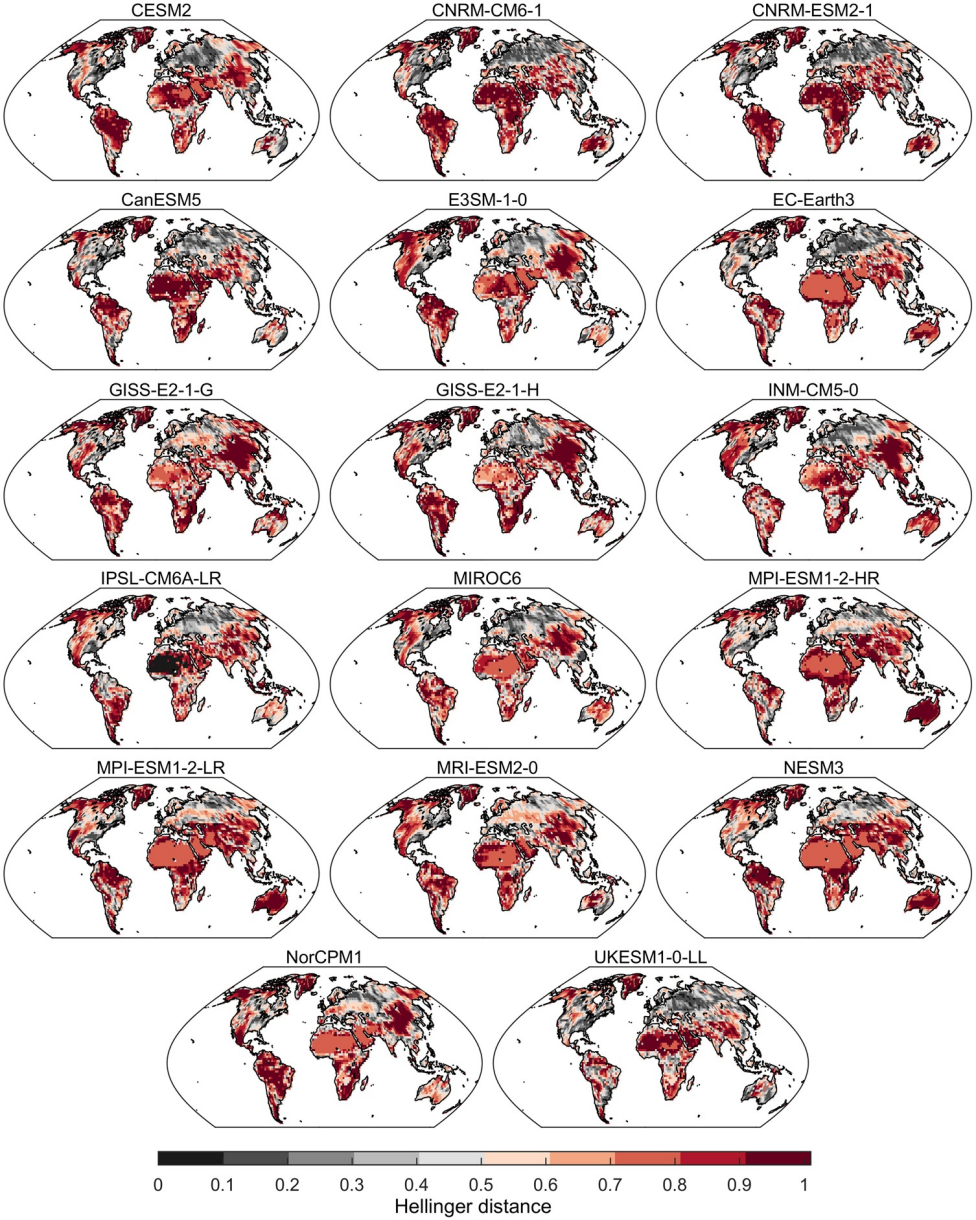
Model performance in reproducing the distribution of low monthly precipitation values defined by the 15% of lowest monthly values. Average Hellinger (H) distance between CRU observations and all runs of each model. Low monthly precipitation distributions of simulations and observations are practically identical as H tends to zero, and as the H distance increases (tending to one) difference between the distributions increases.

We further assessed the model performance by comparing the distribution of low precipitation from all values below the 15‐th percentile in simulations and observations. We calculated the H distance between these empirical distributions and show the average H for each model compared to the CRU observations (Figure 9). High values are observed in Amazonia, desert regions of Africa, middle east, Tibetan plateau, Alaska, Mexico, Australia, and western US indicating poor model performance. In contrast, locations with low H values are found in Europe, eastern US, and equatorial regions of Africa and Asia (Figure 9). This analysis clearly shows that models have considerable bias in low precipitation values and the distribution of low precipitation disagree in many locations.

## 4 Conclusions

Global warming is expected to alter the hydrological cycle (e.g., AghaKouchak et al., 2020; Allen & Ingram, 2002; Bindoff et al., 2014; Zhang et al., 2007). Specifically, drought characteristics are expected to vary considerably over different parts of the world (Andreadis & Lettenmaier, 2006; Sheffield et al., 2009; Trenberth, 2011). It is important to understand such changes for developing adaptation strategies.

Though climate models are useful tools in assessing future changes, apparent biases exist in simulating precipitation. Thus, it is important to evaluate the potential of the models to realistically represent observed extremes; we have more confidence in model projections of future change in models that have a credible representation of historical variability. In this study, we investigated how well the latest climate model simulations from CMIP6 reproduce historical drought characteristics, such as duration and severity. A total of 285 simulations from 17 modeling groups were used to characterize moderate to extreme drought conditions using the 6‐month SPI.

We used three observational global precipitation products, that is, the CRU, GPCC, and UDel, to benchmark simulations. Comparisons were performed with each benchmark product and summarized by combining the results of the three data sets (average performance). Particularly, we estimated the drought duration and severity time series for each grid in observations (three products) and simulations (285 simulations). Basic attributes such as the mean, standard deviation, skewness, and maximum of drought duration were compared, as well as, standard deviation and skewness of drought severity. We devised a novel framework that couples the Hellinger (H) distance with Monte Carlo simulations to quantify the difference between observed and simulated duration probability distributions.

The analysis indicates:

1. Attributes of droughts that appear frequently in observations (close to the mean of drought statistics such as mean, standard deviation, etc.) are well simulated by the models; many climate models show simulated statistical characteristics within ±10% error from observations. Yet infrequent observed characteristics such as low or high values are poorly simulated. In particular, the agreement of simulations with ±10% error in the observed mean, coefficient of variation, skewness and maximum drought duration is 36%, 40%, 16%, and 18%, respectively. For severity, the agreement of runs within this error for standard deviation and skewness is 30% and 11%, respectively.
2. The hypothesis that simulations and observations of monthly precipitation are described by the same distribution cannot be rejected for more than 80% of the grids based on our H distance framework. It should be clear yet that this does not imply acceptance of the hypothesis. We stress that agreement results are affected by the large uncertainty due to small samples, whereas the ±10% error agreement is a stricter measure compared to the H distance approach.
3. No model was clearly better than any other. A spatial pattern of the “best” model (assessed by H distance) is not observed.
4. The variation in drought statistics is higher in the tropics among the 285 simulations compared to other latitudinal zones. This implies that climate models need improvement in capturing patterns causing drought in tropics.
5. Considerable bias in the low precipitation and high H distances reveal dissimilarity in low precipitation (defined as all values below the 15‐th percentile) distribution between observations and simulations. Thus, good model performance in terms of SPI does not imply that low precipitation values are well simulated by the climate models, that is, a systematic bias (shift) in modeled precipitation would be missed by SPI.

Several uncertainties abound in climate model simulations both due to forced and internal variability (Deser et al., 2012; Thompson et al., 2015). Improved knowledge on the range of uncertainties in climate model simulations is important to consider while developing adaptation and mitigation strategies. One key aspect of implicitly incorporating these uncertainties when trying to understand how droughts are projected to change could be the probabilistic model evaluation presented here. By comprehensively examining the statistical agreement of the distributions derived from the models and the observations we can better characterize the agreement or disagreement and potentially pinpoint the model approximations that are causing the latter. Based on such a framework, end users could identify scenarios where simulations are in good agreement with observations. Similarly, climate scientists could develop model usage techniques based on their efficacy to reproduce observations such as weighing schemes not just based on mean and standard deviation (Knutti et al., 2017; Papalexiou et al., 2020).

## Supporting information

Supporting Information S1

## Data Availability

All CMIP6 model simulations and observed data are available at https://esgf-node.llnl.gov/projects/cmip6, https://crudata.uea.ac.uk/cru/data/hrg/, https://opendata.dwd.de/climate_environment/GPCC/html/download_gate.html, and https://psl.noaa.gov/data/gridded/data.UDel_AirT_Precip.html.
