# Supplementary material for: Probabilistic Evaluation of Drought in CMIP6 Simulations
Source: Earths Future. 2021 Oct 11;9(10):e2021EF002150. doi: 10.1029/2021EF002150 (PMC8596413; doi:10.1029/2021EF002150)
Supplement: Supplementary file 1 — Supporting Information S1 [file EFT2-9-e2021EF002150-s001.pdf]

AGU PUBLICATIONS

Earth's Future

Supporting Information for

Probabilistic Evaluation of Drought in CMIP6 Simulations

Simon Michael Papalexiou[1,2,3], Chandra Rupa Rajulapati[2,4], Konstantinos M. Andreadis[5], Efi Foufoula-Georgiou[6,7], Martyn P. Clark[4], Kevin E. Trenberth[8]

[1]Department of Civil Engineering, University of Calgary, Canada
[2]Global Institute for Water Security, Canada
[3]Faculty of Environmental Sciences, Czech University of Life Sciences Prague
[4]Centre for Hydrology, University of Saskatchewan, Saskatoon and Canmore, Canada
[5]Department of Civil and Environmental Engineering, University of Massachusetts Amherst
[6]Department of Civil and Environmental Engineering, University of California, Irvine
[7]Department of Earth System Science, University of California, Irvine
[8]National Center for Atmospheric Research, Boulder, Colorado

Corresponding author: Simon Michael Papalexiou | simon.papalexiou@ucalgary.ca

Supplementary Figures and Tables

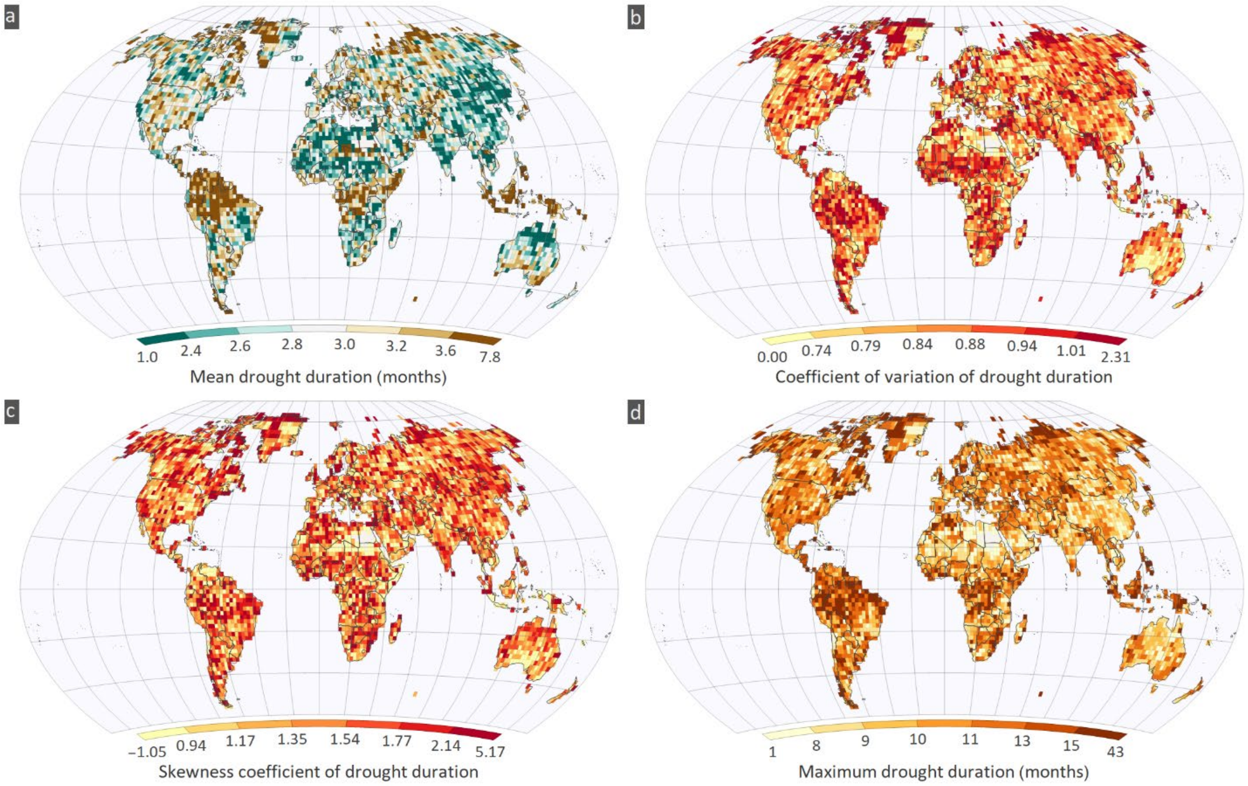

Figure S1. Spatial variation of observed (a) mean (b) coefficient of variation (c) skewness and (d) maximum drought duration for the GPCC dataset for the moderate (SPI ≤ -1) case during 1963-2014.

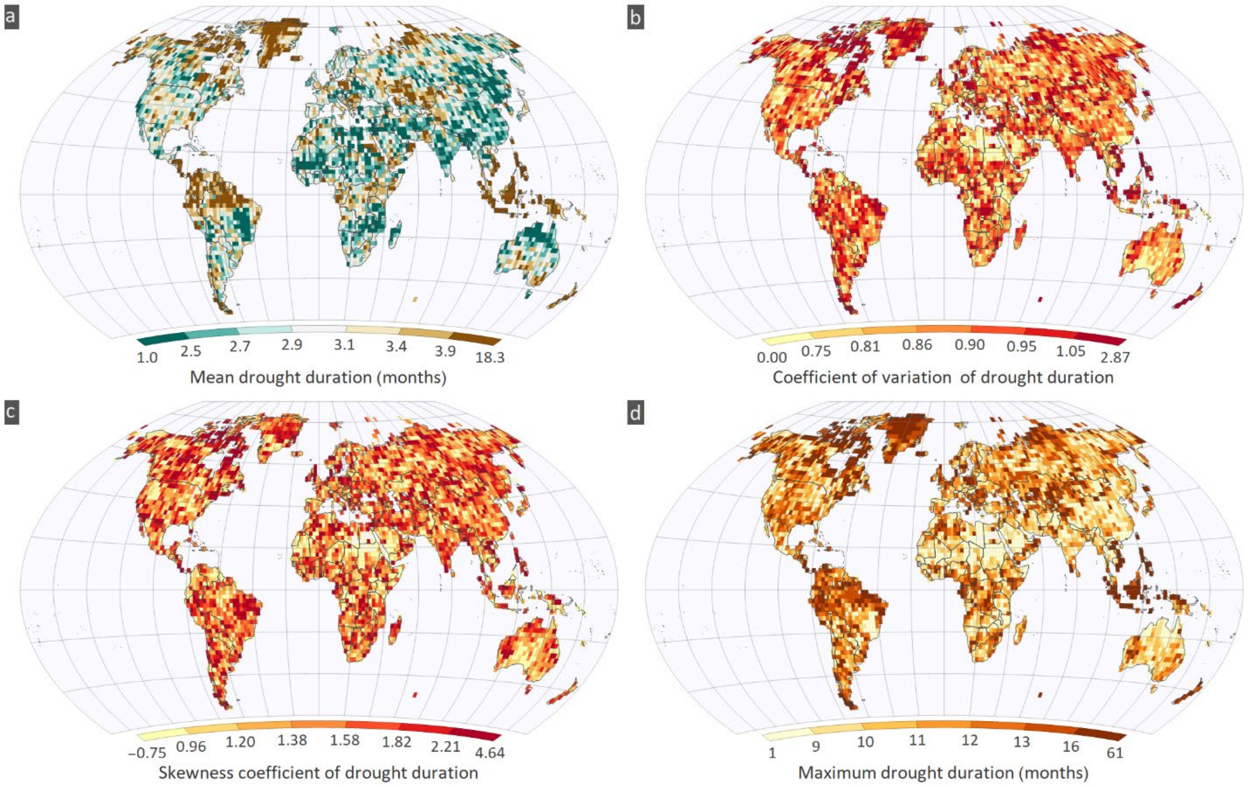

Figure S2. Spatial variation of observed (a) mean (b) coefficient of variation (c) skewness and (d) maximum drought duration for the UDel dataset for the moderate (SPI ≤ -1) case during 1963-2014.

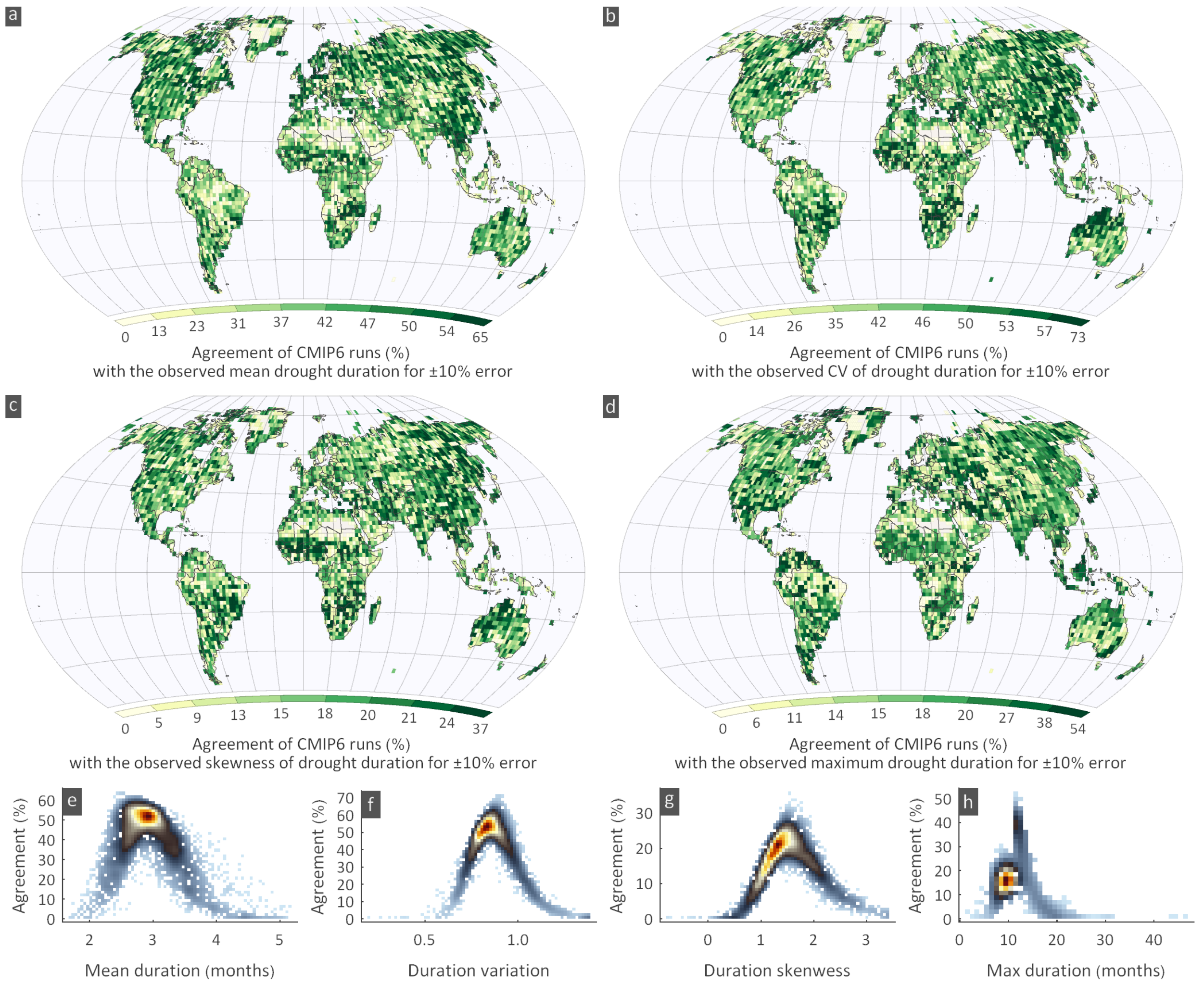

Figure S3. Agreement for drought duration statistics between CMIP6 runs and CRU observations for 1963-2014. (a-d) Spatial variation of agreement in four statistics, (e-h) agreement vs. observed statistics' values.

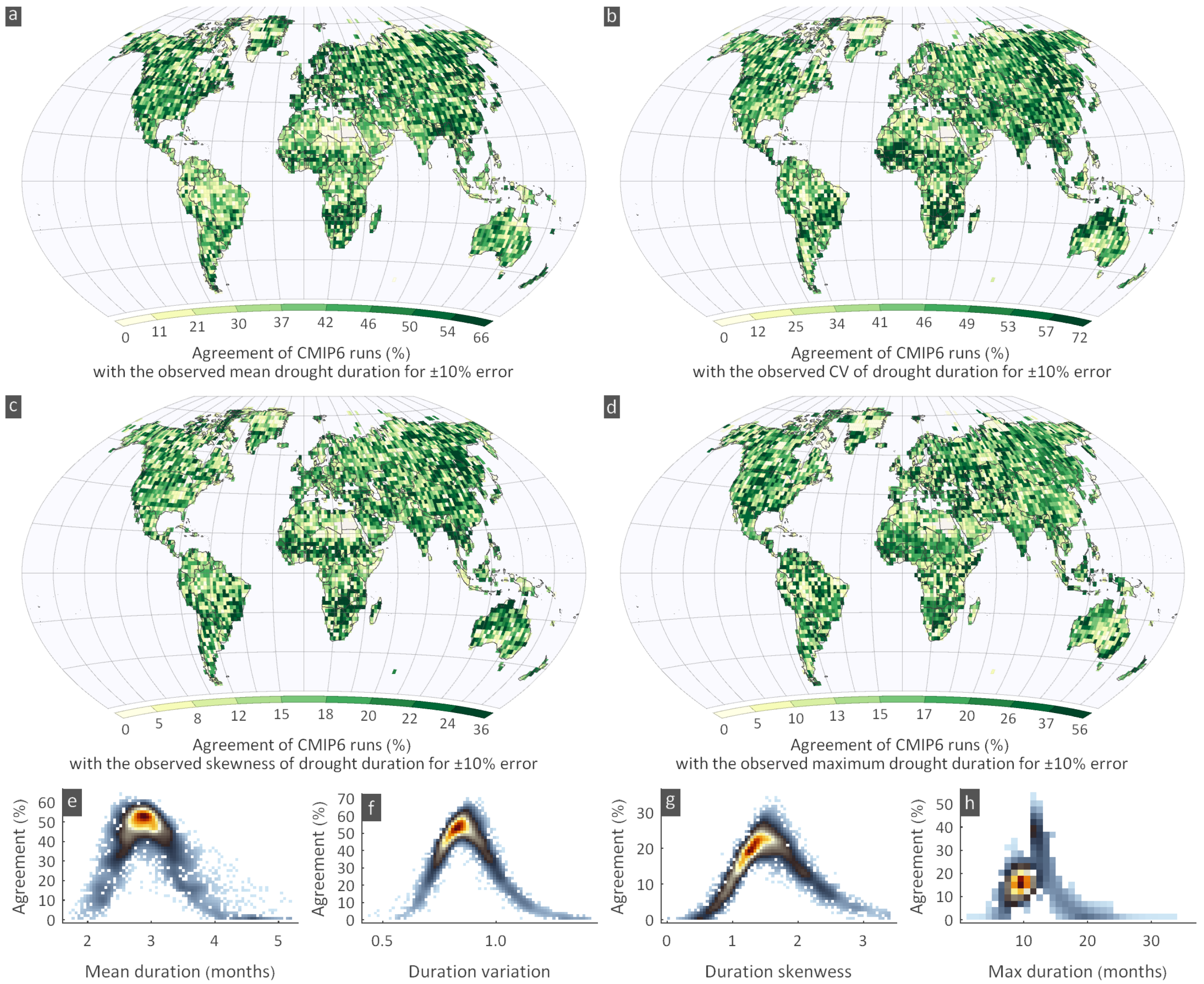

Figure S4. Agreement for drought duration statistics between CMIP6 runs and GPCC observations for 1963-2014. (b-d) Spatial variation of agreement in four statistics, (e-h) agreement vs. observed statistics' values.

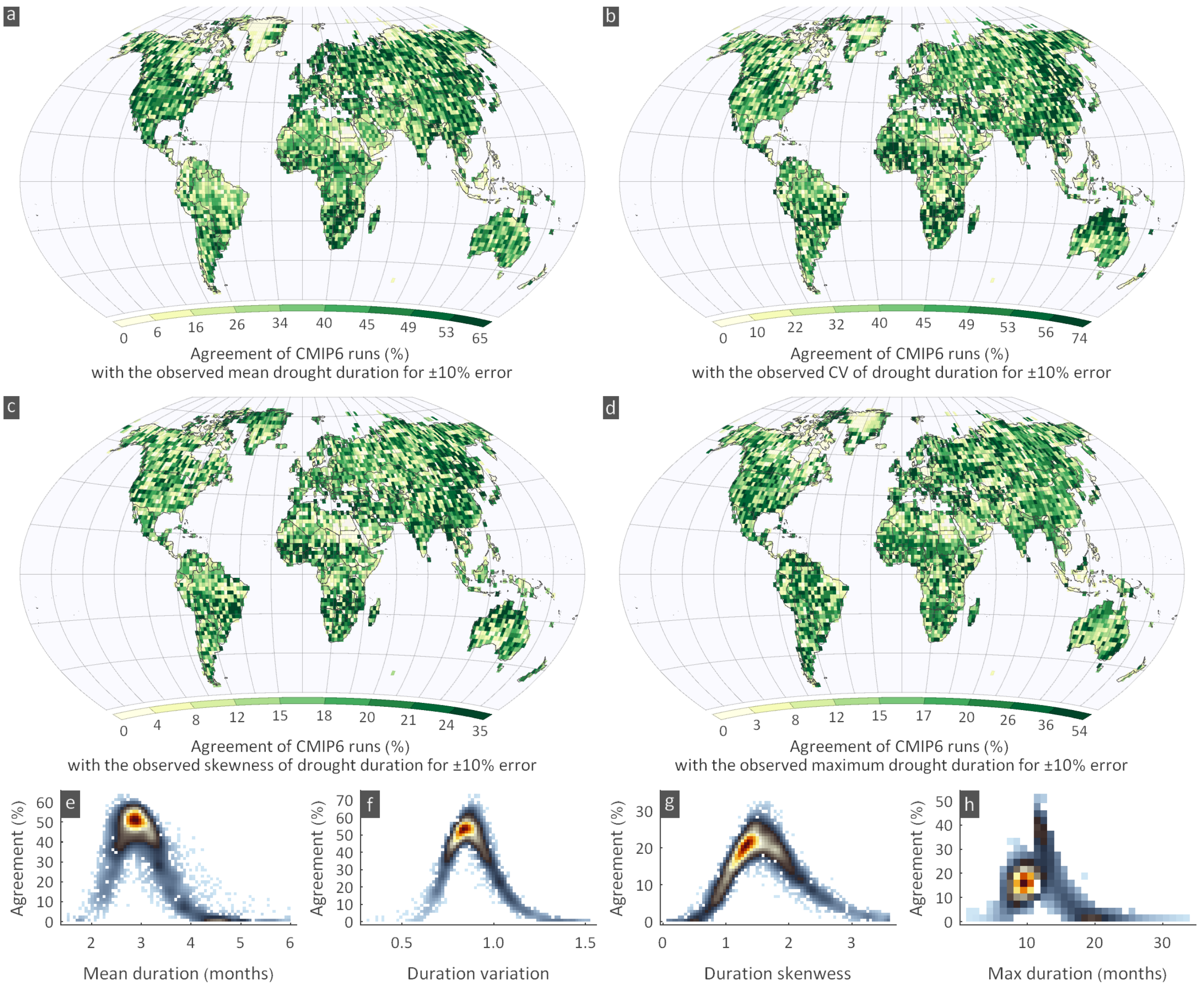

Figure S5. Agreement for drought duration statistics between CMIP6 runs and UDel observations for 1963-2014. (c-d) Spatial variation of agreement in four statistics, (e-h) agreement vs. observed statistics' values.

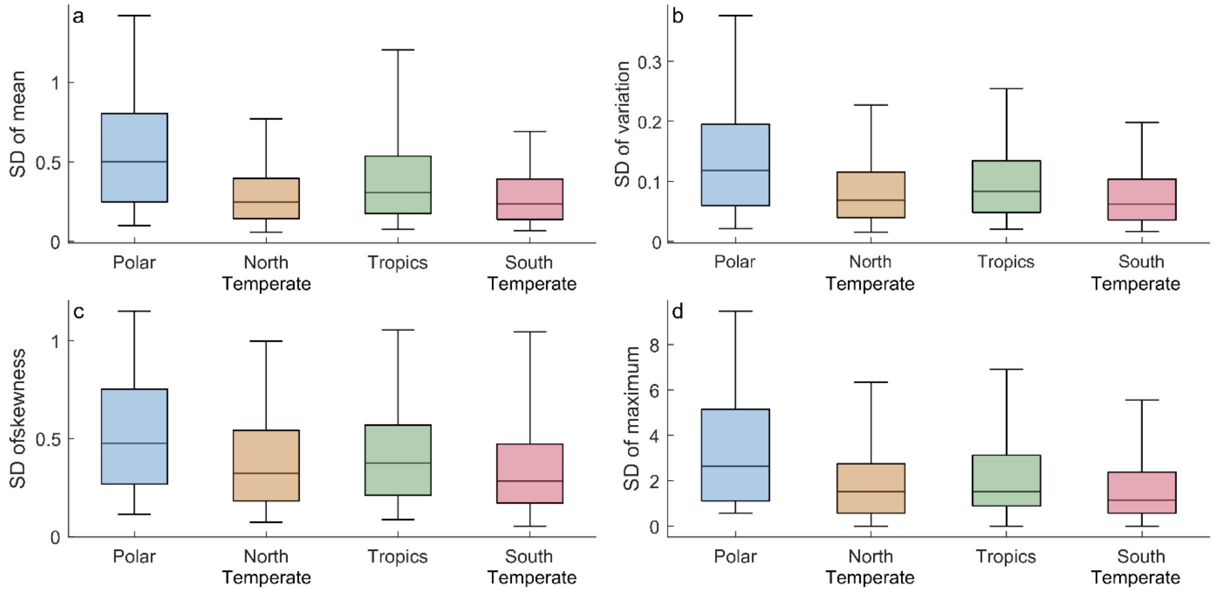

Figure S6. The standard deviation (SD) of a statistic among the three observational datasets is estimated in each grid; then all estimated SDs in each latitudinal zone are depicted as box plots: (a) mean, (b) coefficient of variation, (c) skewness, and (d) maximum of drought durations.

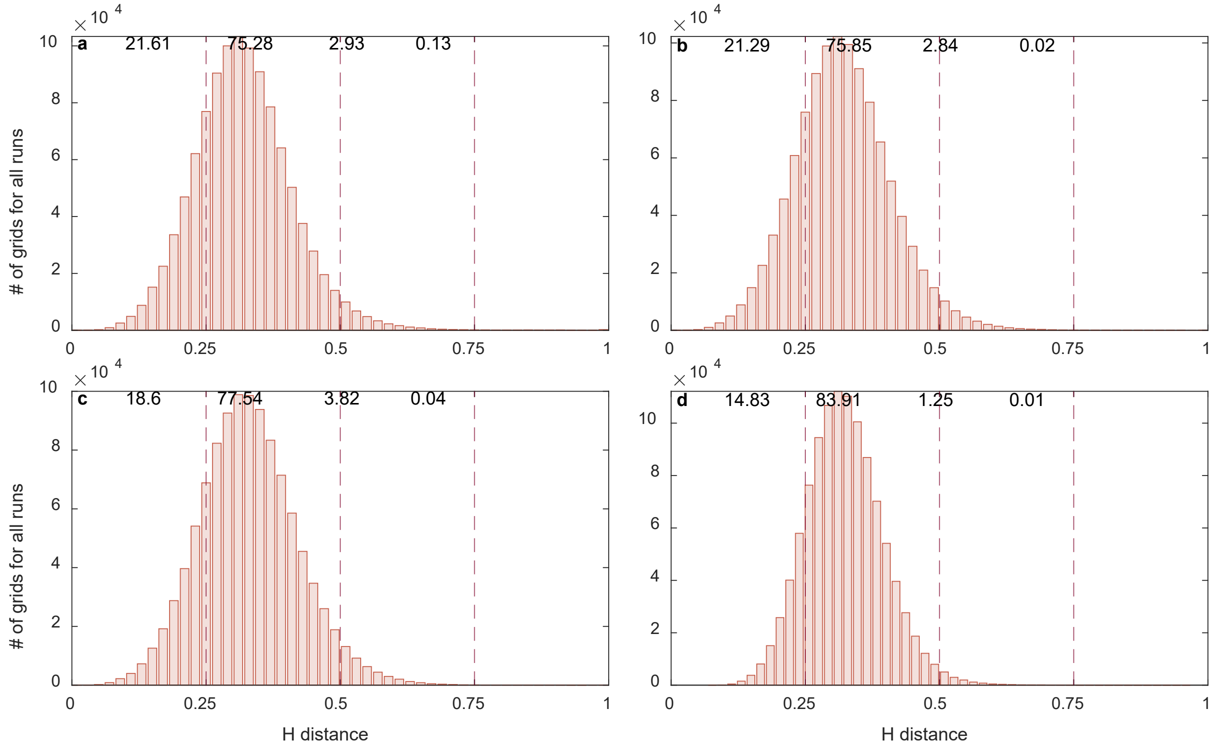

Figure S7. Hellinger distance for all grids and simulations for drought duration using (a) CRU, (b) GPCC and (c) UDel as reference datasets. Subplot (d) shows the average Hellinger distance using the three datasets.

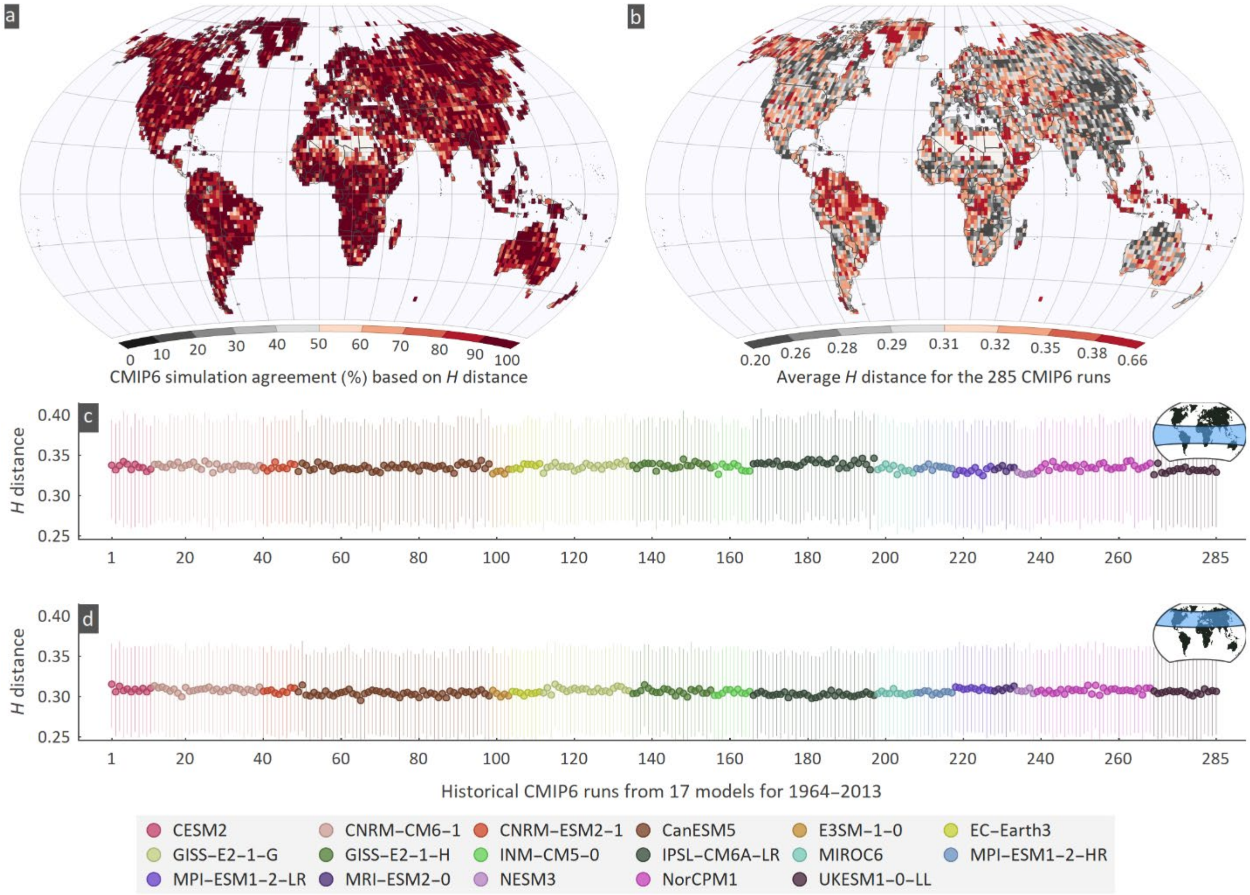

Figure S8. Hellinger (H) distance of CMIP6 runs for the moderate drought duration using CRU dataset. Plots (a) and (b) show the percentage number of simulations with $0 < H < 0.25$ and $0.25 < H < 0.5$ respectively, and (c) and (d) show H distance for the tropical ($23.5°\text{N} - 23.5°\text{S}$) and north temperate ($66.5°\text{N} - 23.5°\text{N}$) zones respectively; Lines represent 50% central H distance values.

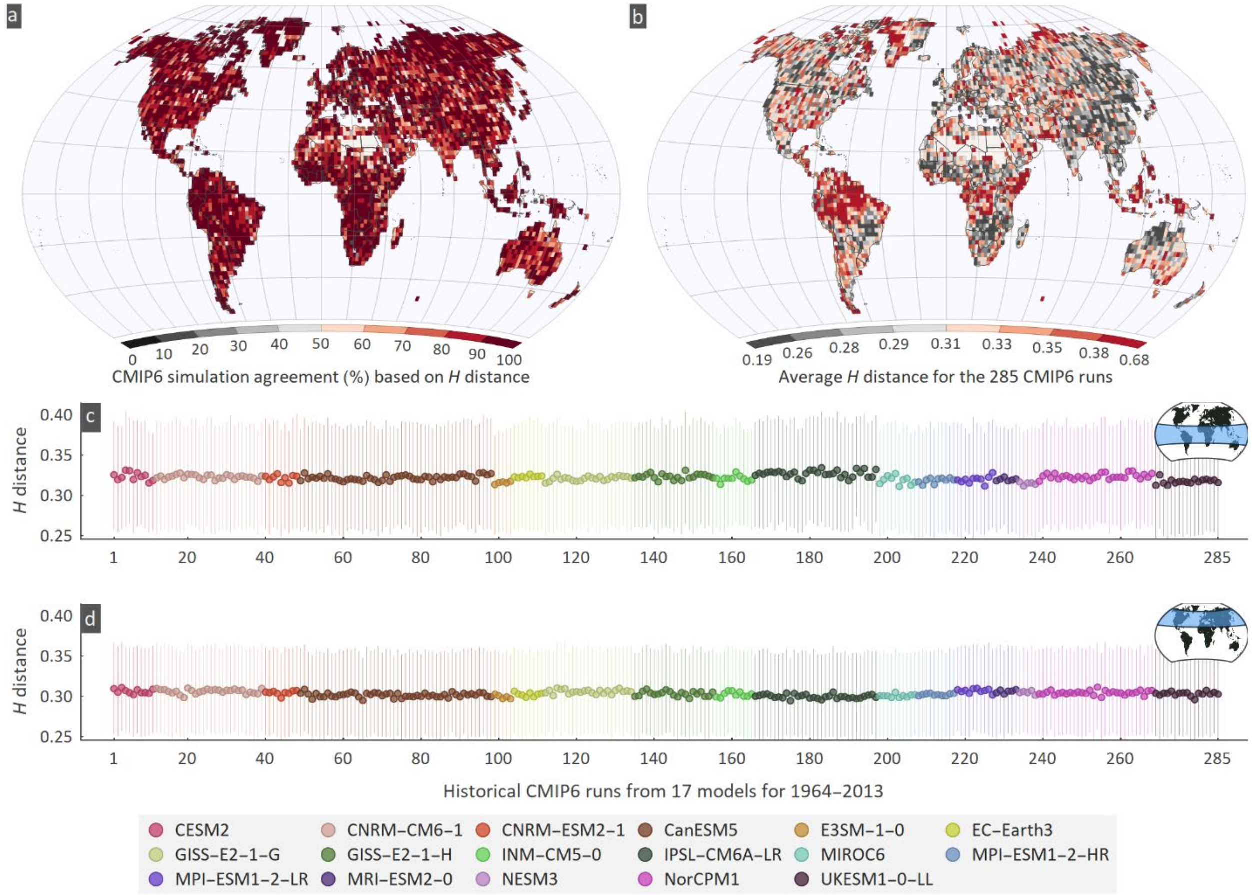

Figure S9. Hellinger (H) distance of CMIP6 runs for the moderate drought duration using GPCC dataset. Plots (e) and (f) show the percentage number of simulations with $0 < H < 0.25$ and $0.25 < H < 0.5$ respectively, and (g) and (h) show H distance for the tropical (23.5°N − 23.5°S) and north temperate (66.5°N − 23.5°N) zones respectively; Lines represent 50% central H distance values.

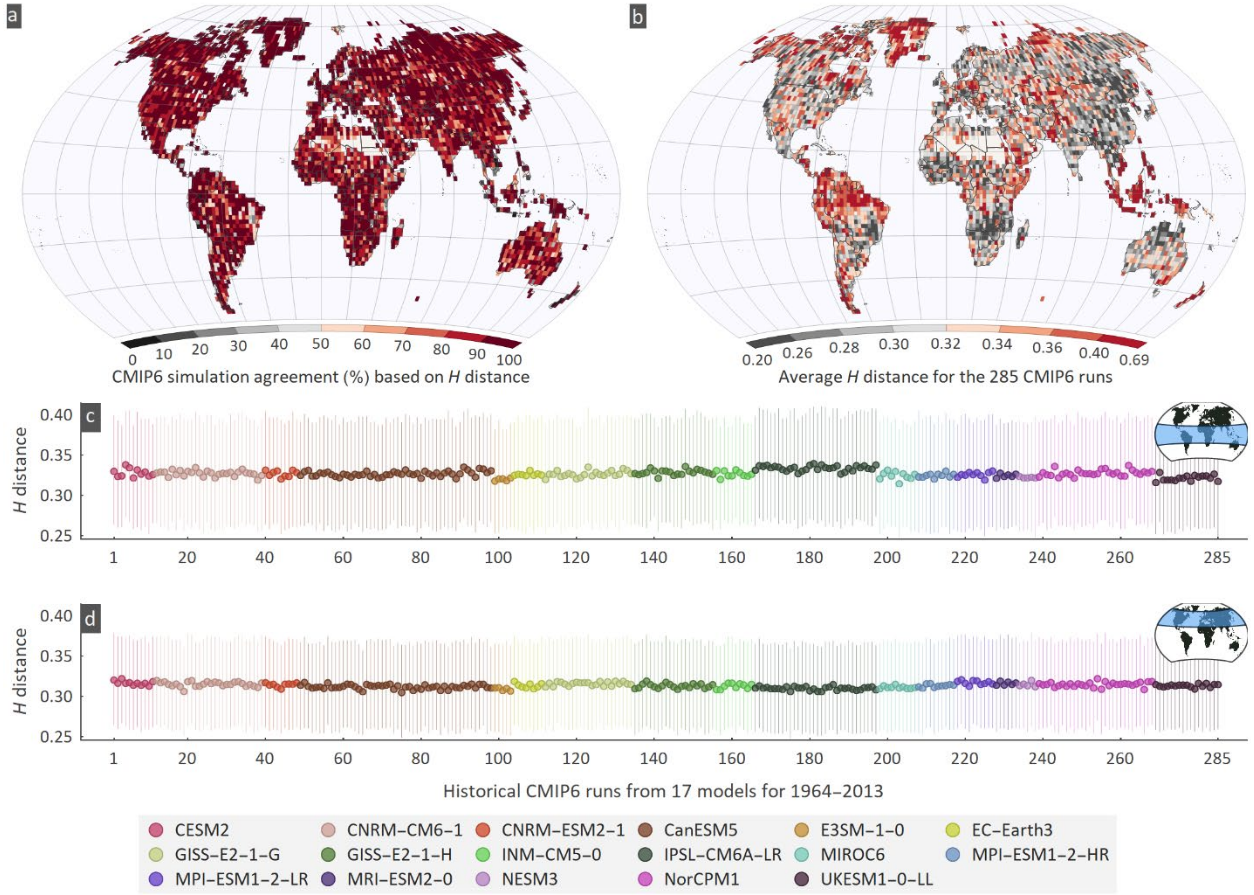

Figure S10. Hellinger (H) distance of CMIP6 runs for the moderate drought duration using UDel dataset. Plots (i) and (j) show the percentage number of simulations with $0 < H < 0.25$ and $0.25 < H < 0.5$ respectively, and (k) and (l) show H distance for the tropical $(23.5°N - 23.5°S)$ and north temperate $(66.5°N - 23.5°N)$ zones respectively; Lines represent 50% central H distance values.

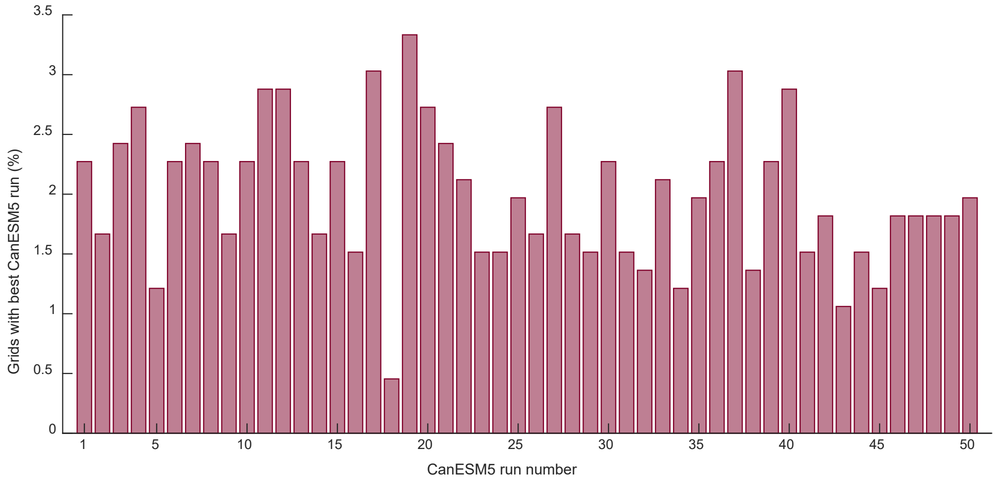

Figure S11. Percentage grids with different variant labels of CanESM5 runs as “best” for drought duration.

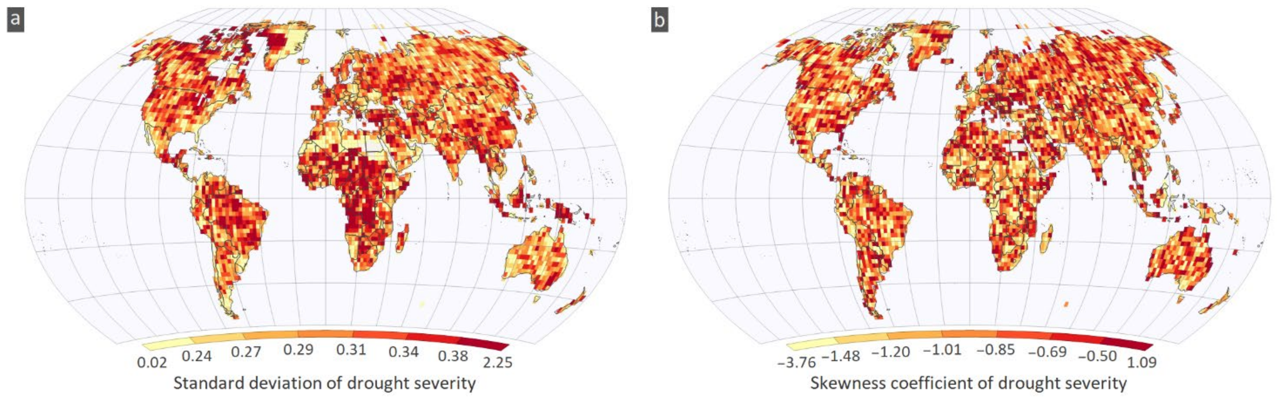

Figure S12. Spatial variability of (a) observed standard deviation and (b) observed skewness of drought severity for GPCC dataset.

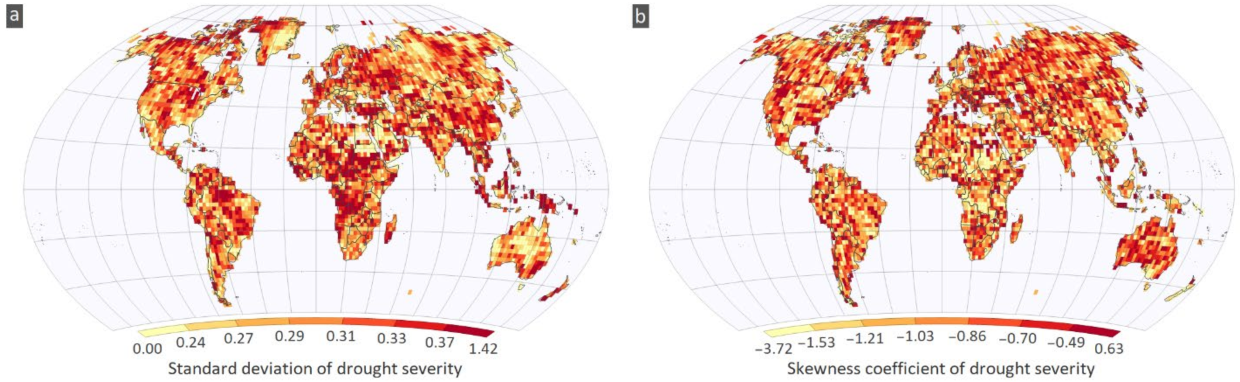

Figure S13. Spatial variability of (a) observed standard deviation and (b) observed skewness of drought severity for UDel dataset .

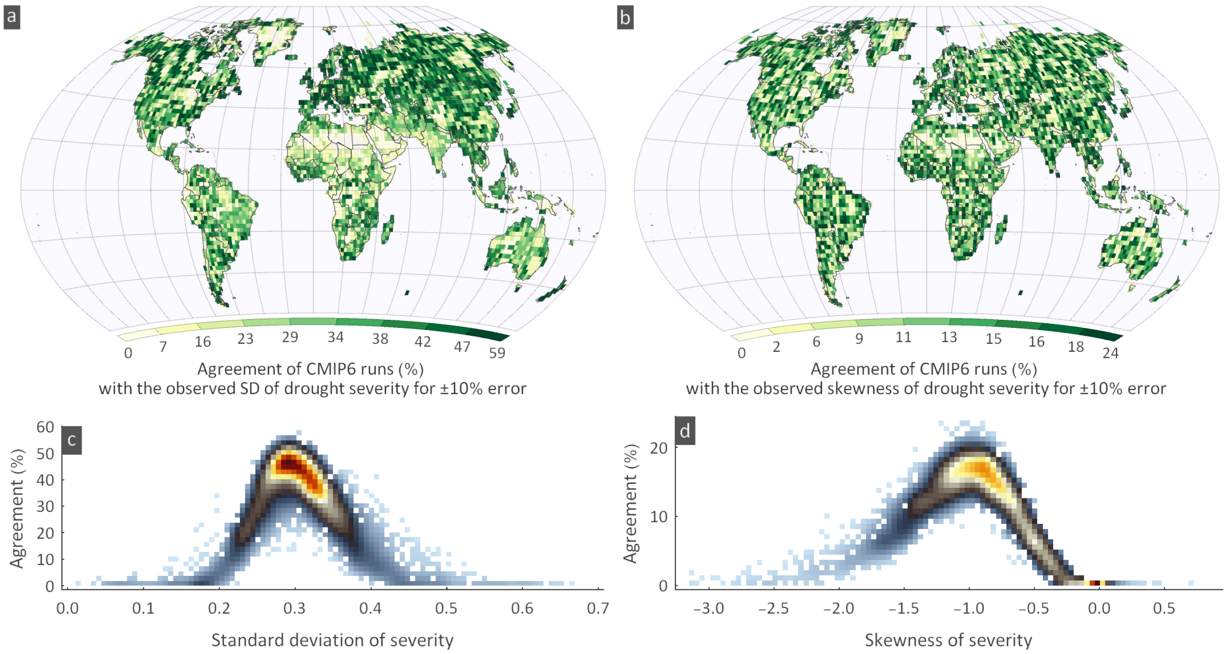

Figure S14. Percentage agreement of CMIP6 simulations with the observed (CRU) (a) Standard Deviation (SD) and (b) skewness. Scatter plots between the percentage agreement of CMIP6 simulations and CRU (c) SD (d) skewness of severity over all land areas for moderate droughts.

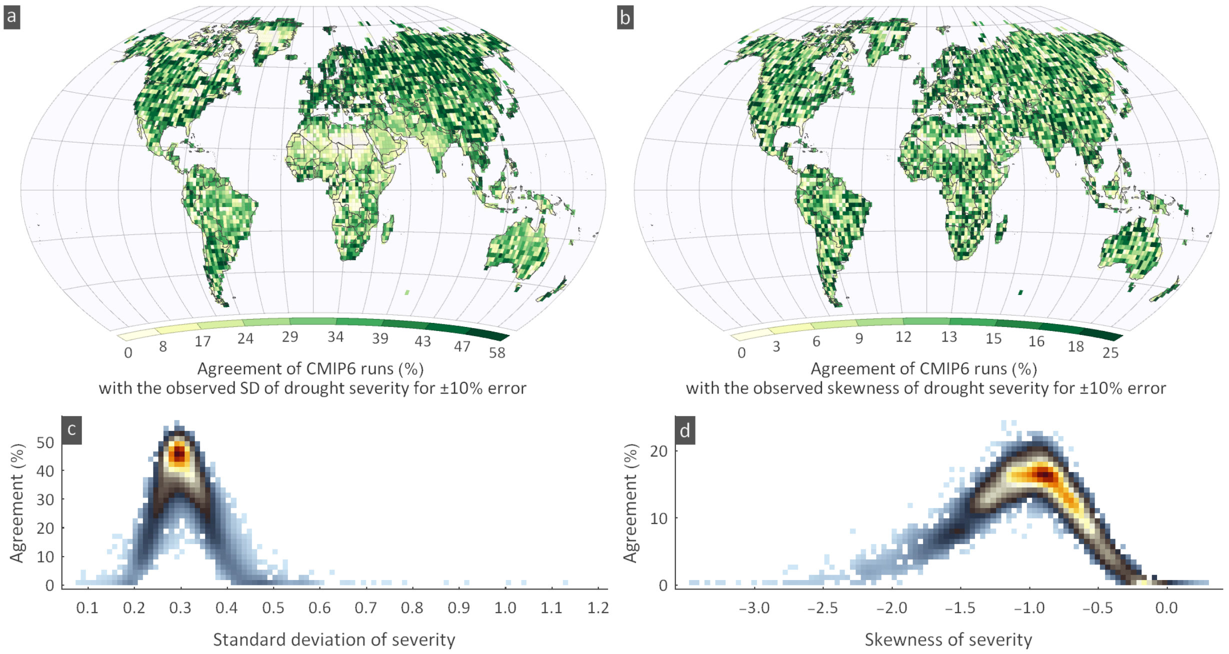

Figure S15. Percentage agreement of CMIP6 simulations with the observed (GPCC) (a) Standard Deviation (SD) and (b) skewness. Scatter plots between the percentage agreement of CMIP6 simulations and GPCC (c) SD (d) skewness of severity over all land areas for moderate droughts.

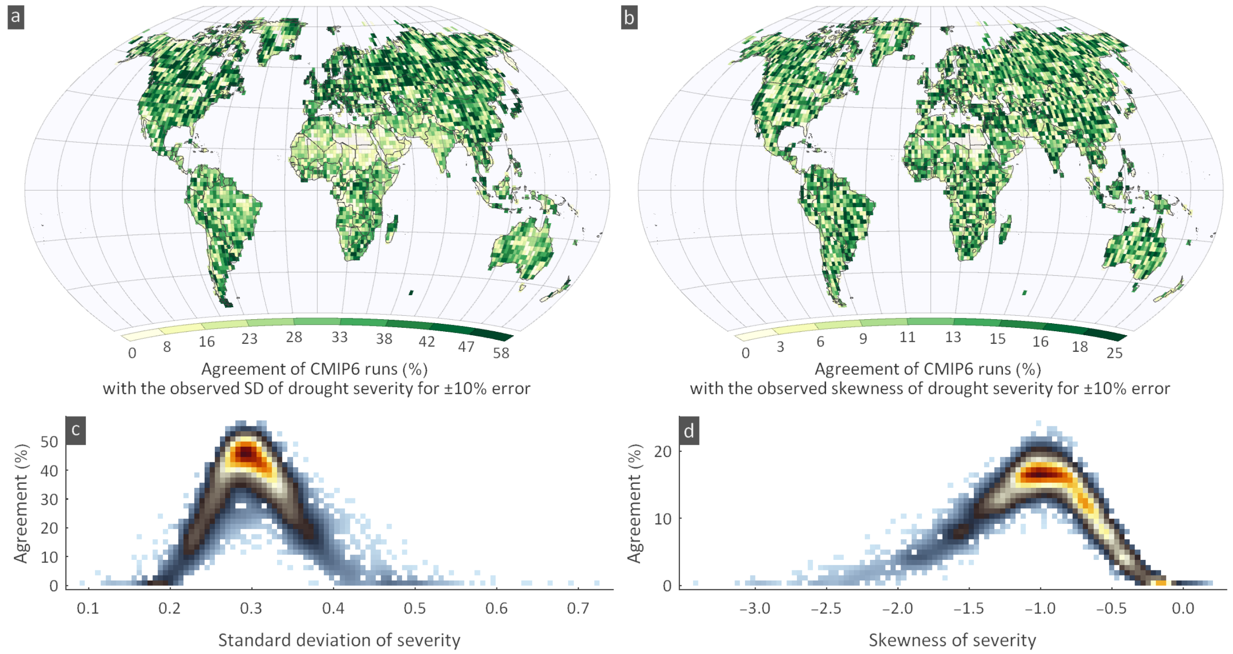

Figure S16. Percentage agreement of CMIP6 simulations with the observed (UDel) (a) Standard Deviation (SD) and (b) skewness. Scatter plots between the percentage agreement of CMIP6 simulations and UDel (c) SD (d) skewness of severity over all land areas for moderate droughts.

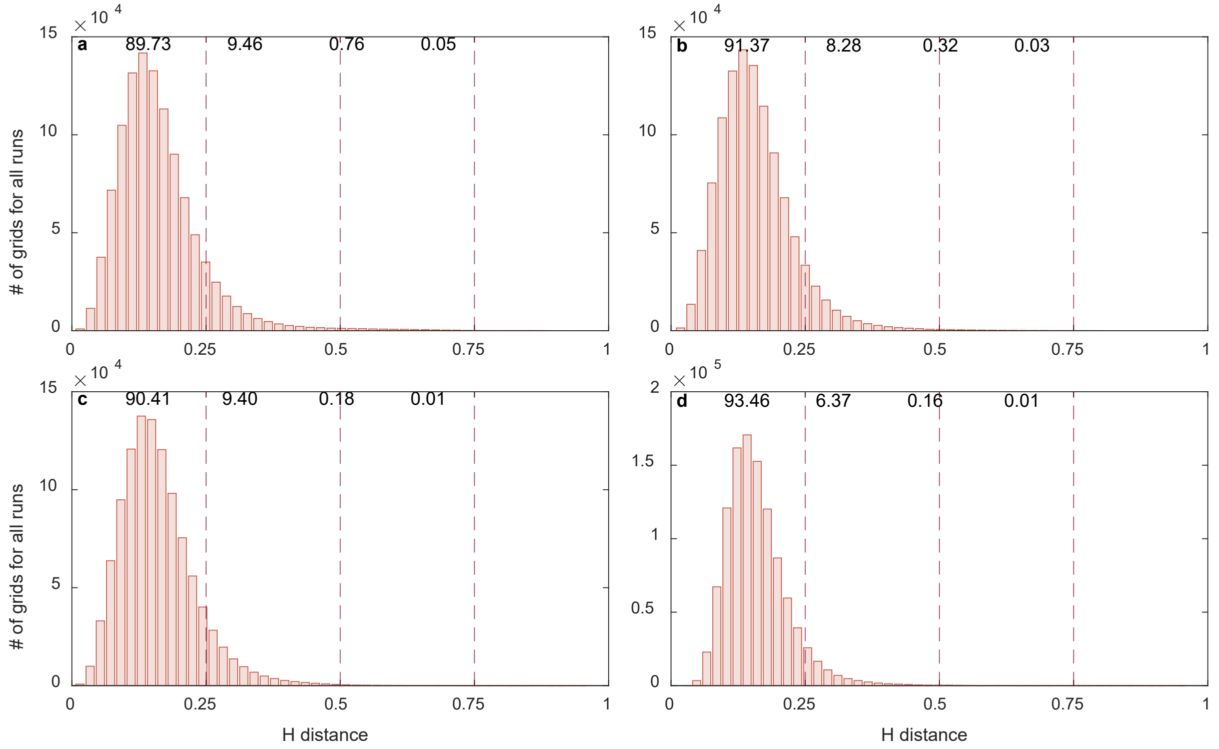

Figure S17. Hellinger distance for all grids and simulations for drought severity using (a) CRU, (b) GPCC and (c) UDel as reference datasets. Subplot (d) shows the average Hellinger distance using the three datasets.

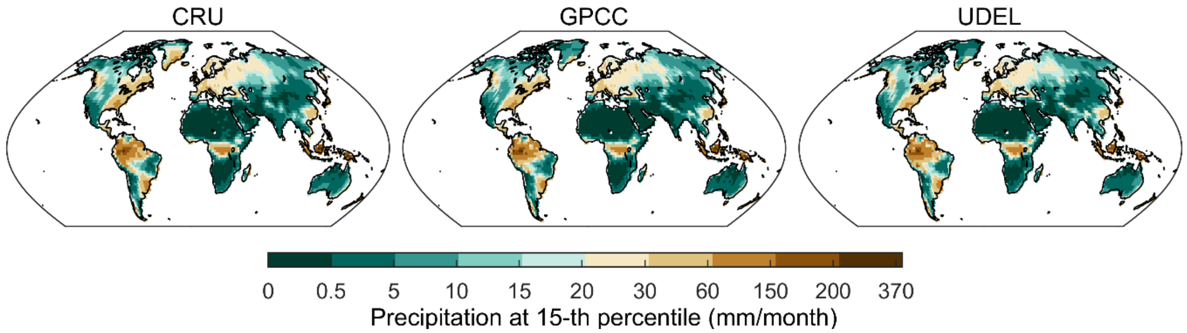

Figure S18. Precipitation (mm/month) at 15.8 percentile for the three observation datasets used in this study.

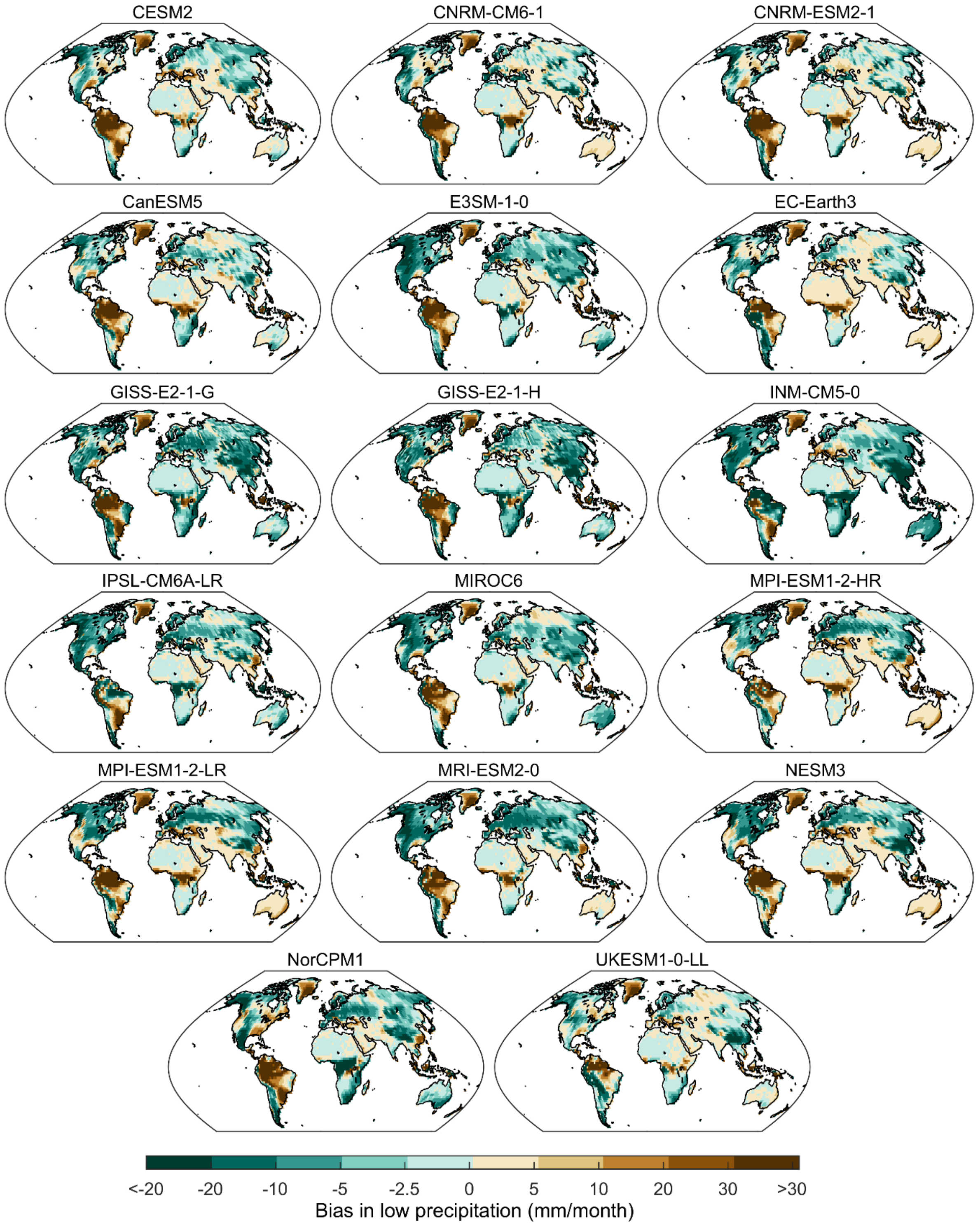

Figure S19. Bias (mm/month) in the low precipitation (defined as the precipitation at 15.8 percentile) among different models using the CRU dataset. Bias is obtained as the difference between observations and the ensemble mean of the model.

Table 1. Details of CMIP6 models and variant labels used in the study (r-realization, initialization, p-physics and f-forcing index).

CMIP6 model name	# of simulations	Variant labels
CESM2	11	r10i1p1f1, r11i1p1f1, r1i1p1f1, r2i1p1f1, r3i1p1f1, r4i1p1f1, r5i1p1f1, r6i1p1f1, r7i1p1f1, r8i1p1f1, r9i1p1f1
CNRM-CM6-1	28	r10i1p1f2, r11i1p1f2, r12i1p1f2, r13i1p1f2, r14i1p1f2, r15i1p1f2, r16i1p1f2, r17i1p1f2, r18i1p1f2, r19i1p1f2, r1i1p1f2, r21i1p1f2, r22i1p1f2, r24i1p1f2, r25i1p1f2, r26i1p1f2, r27i1p1f2, r28i1p1f2, r29i1p1f2, r2i1p1f2, r30i1p1f2, r3i1p1f2, r4i1p1f2, r5i1p1f2, r6i1p1f2, r7i1p1f2, r8i1p1f2, r9i1p1f2
CNRM-ESM2-1	9	r10i1p1f2, r1i1p1f2, r2i1p1f2, r3i1p1f2, r4i1p1f2, r5i1p1f2, r7i1p1f2, r8i1p1f2, r9i1p1f2
CanESM5	50	r10i1p1f1, r10i1p2f1, r11i1p1f1, r11i1p2f1, r12i1p1f1, r12i1p2f1, r13i1p1f1, r13i1p2f1, r14i1p1f1, r14i1p2f1, r15i1p1f1, r15i1p2f1, r16i1p1f1, r16i1p2f1, r17i1p1f1, r17i1p2f1, r18i1p1f1, r18i1p2f1, r19i1p1f1, r19i1p2f1, r1i1p1f1, r1i1p2f1, r20i1p1f1, r20i1p2f1, r21i1p1f1, r21i1p2f1, r22i1p1f1, r22i1p2f1, r23i1p1f1, r23i1p2f1, r24i1p1f1, r24i1p2f1, r25i1p1f1, r25i1p2f1, r2i1p1f1, r2i1p2f1, r3i1p1f1, r3i1p2f1, r4i1p1f1, r4i1p2f1, r5i1p1f1, r5i1p2f1, r6i1p1f1, r6i1p2f1, r7i1p1f1, r7i1p2f1, r8i1p1f1, r8i1p2f1, r9i1p1f1, r9i1p2f1
E3SM-1-0	5	r1i1p1f1, r2i1p1f1, r3i1p1f1, r4i1p1f1, r5i1p1f1
EC-Earth3	8	r12i1p1f1, r14i1p1f1, r16i1p1f1, r17i1p1f1, r18i1p1f1, r22i1p1f1, r24i1p1f1, r7i1p1f1
GISS-E2-1-G	23	r101i1p1f1, r102i1p1f1, r10i1p1f1, r10i1p1f2, r1i1p1f1, r1i1p1f2, r2i1p1f1, r2i1p1f2, r3i1p1f1, r3i1p1f2, r4i1p1f1, r4i1p1f2, r5i1p1f1, r5i1p1f2, r6i1p1f1, r6i1p1f2, r6i1p3f1, r7i1p1f1, r7i1p1f2, r8i1p1f1, r8i1p1f2, r9i1p1f1, r9i1p1f2
GISS-E2-1-H	21	r10i1p1f1, r1i1p1f1, r1i1p1f2, r1i1p5f1, r2i1p1f1, r2i1p1f2, r2i1p5f1, r3i1p1f1, r3i1p1f2, r3i1p3f1, r3i1p5f1, r4i1p1f1, r4i1p1f2, r4i1p3f1, r5i1p1f1, r5i1p1f2, r5i1p3f1, r6i1p1f1, r7i1p1f1, r8i1p1f1, r9i1p1f1
INM-CM5-0	10	r10i1p1f1, r1i1p1f1, r2i1p1f1, r3i1p1f1, r4i1p1f1, r5i1p1f1, r6i1p1f1, r7i1p1f1, r8i1p1f1, r9i1p1f1

IPSL-CM6A-LR	32	r10i1p1f1, r11i1p1f1, r12i1p1f1, r13i1p1f1, r14i1p1f1, r15i1p1f1, r16i1p1f1, r17i1p1f1, r18i1p1f1, r19i1p1f1, r1i1p1f1, r20i1p1f1, r21i1p1f1, r22i1p1f1, r23i1p1f1, r24i1p1f1, r25i1p1f1, r26i1p1f1, r27i1p1f1, r28i1p1f1, r29i1p1f1, r2i1p1f1, r30i1p1f1, r31i1p1f1, r32i1p1f1, r3i1p1f1, r4i1p1f1, r5i1p1f1, r6i1p1f1, r7i1p1f1, r8i1p1f1,
MIROC6	10	r10i1p1f1, r1i1p1f1, r2i1p1f1, r3i1p1f1, r4i1p1f1, r5i1p1f1, r6i1p1f1, r7i1p1f1, r8i1p1f1, r9i1p1f1
MPI-ESM1-2-HR	10	r10i1p1f1, r1i1p1f1, r2i1p1f1, r3i1p1f1, r4i1p1f1, r5i1p1f1, r6i1p1f1, r7i1p1f1, r8i1p1f1, r9i1p1f1
MPI-ESM1-2-LR	10	r10i1p1f1, r1i1p1f1, r2i1p1f1, r3i1p1f1, r4i1p1f1, r5i1p1f1, r6i1p1f1, r7i1p1f1, r8i1p1f1, r9i1p1f1
MRI-ESM2-0	6	r1i1p1f1, r1i2p1f1, r2i1p1f1, r3i1p1f1, r4i1p1f1, r5i1p1f1
NESM3	5	r1i1p1f1, r2i1p1f1, r3i1p1f1, r4i1p1f1, r5i1p1f1
NorCPM1	30	r10i1p1f1, r11i1p1f1, r12i1p1f1, r13i1p1f1, r14i1p1f1, r15i1p1f1, r16i1p1f1, r17i1p1f1, r18i1p1f1, r19i1p1f1, r1i1p1f1, r20i1p1f1, r21i1p1f1, r22i1p1f1, r23i1p1f1, r24i1p1f1, r25i1p1f1, r26i1p1f1, r27i1p1f1, r28i1p1f1, r29i1p1f1, r2i1p1f1, r30i1p1f1, r3i1p1f1, r4i1p1f1, r5i1p1f1, r6i1p1f1, r7i1p1f1, r8i1p1f1, r9i1p1f1
UKESM1-0-LL	17	r10i1p1f2, r11i1p1f2, r12i1p1f2, r13i1p1f2, r16i1p1f2, r17i1p1f2, r18i1p1f2, r19i1p1f2, r1i1p1f2, r2i1p1f2, r3i1p1f2, r4i1p1f2, r5i1p1f3, r6i1p1f3, r7i1p1f3, r8i1p1f2, r9i1p1f2
